# Assessment of Cannabidiol and Δ9-Tetrahydrocannabiol in Mouse Models of Medulloblastoma and Ependymoma

**DOI:** 10.3390/cancers13020330

**Published:** 2021-01-18

**Authors:** Clara Andradas, Jacob Byrne, Mani Kuchibhotla, Mathew Ancliffe, Anya C. Jones, Brooke Carline, Hilary Hii, Alexandra Truong, Lisa C. D. Storer, Timothy A. Ritzmann, Richard G. Grundy, Nicholas G. Gottardo, Raelene Endersby

**Affiliations:** 1 Brain Tumour Research Program, Telethon Kids Institute, Nedlands, WA 6009, Australia; Jacob.Byrne@telethonkids.org.au (J.B.); Mani.Kuchibhotla@telethonkids.org.au (M.K.); Mathew.Ancliffe@telethonkids.org.au (M.A.); Brooke.Carline@telethonkids.org.au (B.C.); Hilary.Hii@telethonkids.org.au (H.H.); 22725235@student.uwa.edu.au (A.T.); Nick.Gottardo@health.wa.gov.au (N.G.G.); 2 Centre for Child Health Research, University of Western Australia, Nedlands, WA 6009, Australia; 3 School of Veterinary and Life Sciences, Murdoch University, Murdoch, WA 6150, Australia; 4 Telethon Kids Cancer Centre, Telethon Kids Institute, Nedlands, WA 6009, Australia; Anya.Jones@telethonkids.org.au; 5 Children’s Brain Tumour Research Centre, University of Nottingham, Nottingham NG7 2RD, UK; lisastorer11@gmail.com (L.C.D.S.); Timothy.Ritzmann1@nottingham.ac.uk (T.A.R.); richard.grundy@nottingham.ac.uk (R.G.G.); 6 Department of Paediatric and Adolescent Oncology/Haematology, Perth Children’s Hospital, Nedlands, WA 6009, Australia

**Keywords:** medulloblastoma, ependymoma, cannabinoid, medical cannabis, Δ9-tetrahydrocannabinol, THC, CBD, cannabidiol, pediatric oncology, childhood cancer

## Abstract

**Simple Summary:**

Phytocannabinoids Δ9-tetrahydrocannabinol (THC) and cannabidiol (CBD) have been demonstrated to exhibit anti-cancer activity in preclinical models of brain cancer leading to new clinical trials for adults with glioblastoma. We describe here the first report that has investigated a role for THC and CBD in pediatric brain cancer. Cannabinoids had cytotoxic activity against medulloblastoma and ependymoma cells in vitro, functioning in part through the inhibition of cell cycle progression and the induction of autophagy. Despite these effects in vitro, when tested in orthotopic mouse models of medulloblastoma or ependymoma, no impact on animal survival was observed. Furthermore, cannabinoids neither enhanced nor impaired conventional chemotherapy in a medulloblastoma mouse model. These data show that while THC and CBD do have some effects on medulloblastoma and ependymoma cells, are well tolerated, and have minimal adverse effects, they do not appear to elicit any survival benefit in preclinical models of pediatric brain cancer.

**Abstract:**

Children with medulloblastoma and ependymoma are treated with a multidisciplinary approach that incorporates surgery, radiotherapy, and chemotherapy; however, overall survival rates for patients with high-risk disease remain unsatisfactory. Data indicate that plant-derived cannabinoids are effective against adult glioblastoma; however, preclinical evidence supporting their use in pediatric brain cancers is lacking. Here we investigated the potential role for Δ9-tetrahydrocannabinol (THC) and cannabidiol (CBD) in medulloblastoma and ependymoma. Dose-dependent cytotoxicity of medulloblastoma and ependymoma cells was induced by THC and CBD in vitro, and a synergistic reduction in viability was observed when both drugs were combined. Mechanistically, cannabinoids induced cell cycle arrest, in part by the production of reactive oxygen species, autophagy, and apoptosis; however, this did not translate to increased survival in orthotopic transplant models despite being well tolerated. We also tested the combination of cannabinoids with the medulloblastoma drug cyclophosphamide, and despite some in vitro synergism, no survival advantage was observed in vivo. Consequently, clinical benefit from the use of cannabinoids in the treatment of high-grade medulloblastoma and ependymoma is expected to be limited. This study emphasizes the importance of preclinical models in validating therapeutic agent efficacy prior to clinical trials, ensuring that enrolled patients are afforded the most promising therapies available.

## 1. Introduction

Pediatric medulloblastoma and ependymoma represent the first and third most common childhood brain malignancies, respectively [1]. Current up-front treatment for medulloblastoma involves maximal safe surgical resection, followed by craniospinal irradiation and a combination of chemotherapeutic agents such as tubulin inhibitors and DNA alkylators [2]. Extensive transcriptomic analyses have categorized medulloblastoma into four molecular subgroups: WNT, SHH, Group 3, and Group 4, that can be further stratified into 13 molecular subtypes [3]. Each subtype warrants different management regimes to maximize tumor response to treatment due to genetic differences, although these are yet to be defined. Currently, the poorest survival rates are associated with *MYC*-amplified Group 3 or *TP53*-mutated SHH disease, highlighting the urgency for the development of novel therapies to improve survival rates for these medulloblastoma subtypes.

Ependymoma arises from the ependymal cells lining the ventricular walls of the central nervous system (CNS) and accounts for 6–12% of all intracranial tumors in children, with up to 30% diagnosed in those younger than three years [4]. Contemporary standard-of-care treatment for ependymoma patients is surgery followed by focal radiotherapy [5]. Despite this, 40–60% of these tumors will recur at the primary site of disease or with metastatic spread [4,6]. The role of chemotherapy in this disease remains uncertain and is currently being investigated in several international clinical trials (clinicaltrials.gov identifiers: NCT01096368 and NCT02265770) [7,8]. Although no chemotherapeutic regimen has proven clinically beneficial in the treatment of pediatric ependymoma to date, results from ACNS0831 suggest that some patients may benefit from maintenance chemotherapy [9]. Complicating the current lack of safe and efficacious therapies, ependymal malignancies with similar histological grades exhibit genetic heterogeneity, leading to a diverse range of patient outcomes despite identical treatment strategies [10]. On the basis of transcriptomics and DNA methylation patterns, ependymoma has also been delineated into multiple subgroups, of which posterior fossa A ependymoma (EPN_PFA) and *C11orf95* fusion-positive ependymoma (formerly EPN_RELA, and recently renamed due to the identification of other fusion partners for *C11orf95*) predominate in children [11,12]

While our understanding of the molecular pathogenesis of these diseases has improved dramatically in the last three decades, this knowledge has not translated to increased patient survival. Amongst survivors, current treatments can cause debilitating side effects, including untreatable secondary malignancies [13], cognitive dysfunction, cardiotoxicity, myelosuppression, renal toxicity, and endocrine problems [14]. Therefore, improved clinical outcomes are dependent on the identification of efficacious therapies that not only increase survival but reduce treatment-related side effects.

Numerous research studies since the late 1990s have contributed to a growing body of evidence demonstrating that various cannabinoids have anti-cancer effects. These include studies performed in a wide variety of experimental models of cancer, ranging from cancer cell lines in culture to genetically engineered mice, and covering several cancers including lymphomas and adult brain tumors [15]. Δ9-tetrahydrocannabinol (THC) is one of the primary compounds that can be isolated from the plant *Cannabis sativa L* and is known to exert a broad range of biological and psychoactive effects. THC mimics the actions of endocannabinoids, a family of lipid-based signaling molecules, by binding to and activating cannabinoid receptors type 1 (CB_1_R) and type 2 (CB_2_R) [16,17]. These two G-protein coupled receptors are primarily expressed in cells within the CNS and immune system, respectively. Cannabidiol (CBD) is another highly abundant cannabinoid in *Cannabis sativa L* extracts that has been demonstrated to also exert biological effects in mammals; however, unlike THC, CBD has low affinity for CB_1_R and CB_2_R [18]. Recent studies have shown that CBD instead targets several other G-protein coupled receptors, such as GPR55, GPR18, and 5-HT1A [19,20], and transient receptor potential (TRP) channels such as TRPV1 and TRPV2 [21,22]; however, its mechanism of action in mammals is yet to be fully elucidated.

Research into the effects of THC and CBD in different types of cancer overwhelmingly show that THC and CBD induce cancer cell death [23]. The more intriguing brain tumor-related studies have been in mouse models of glioblastoma (the most common adult brain cancer) and showed that both THC and CBD improved animal survival when administered to mice in combination with the standard of care chemotherapy temozolomide [24,25]. Furthermore, when glioma cells were pre-treated with THC or CBD, either in vitro or in vivo, this sensitized the cancer cells to radiation-induced death and prolonged survival of mice [26]. Mechanistically, the effects of cannabinoids on glioblastoma cells are mediated by the inhibition of proliferation and the induction of cell death via autophagy and apoptotic mechanisms [27,28,29].

There is no existing data on the effect of these agents in pediatric brain tumor models in vitro or in vivo. Some anecdotal reports can be found describing the benefits of medicinal cannabis for these patients, but assessment of these is challenging, because the dosages and exact components of the plant extracts used have not been comprehensively documented (as would be done in a conventional clinical trial). As such, any data from these patients is unreliable. With the increasing availability of medicinal cannabis, there is a growing demand from patients, parents, and physicians for better information describing the safety and efficacy of cannabinoids in pediatric brain tumors [30,31,32], although reports describing the proportion of pediatric oncology patients actively seeking or using medical cannabis are scarce. A recent report from the Children’s Hospital of Minnesota stated that hope for an anti-tumor effect was the major reason parents sought medical cannabis for children with brain cancer despite a lack of evidence demonstrating such an effect [32]. To address these gaps in the literature and to help identify novel anti-cancer agents with better safety profiles for the treatment of these diseases, our study aimed to investigate the anti-cancer efficacy and mechanisms of action for THC and CBD in preclinical models of pediatric medulloblastoma and ependymoma.

## 2. Results

### 2.1. Cannabinoid Receptors Are Expressed in Pediatric Medulloblastoma and Ependymoma

**Figure 1:**
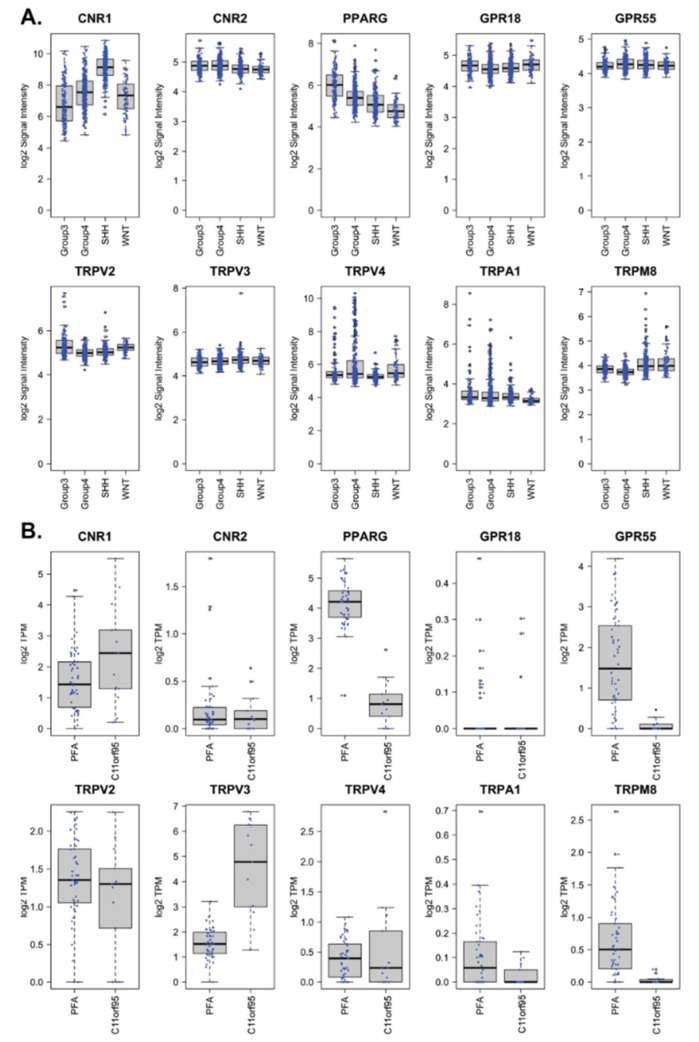
Cannabinoid receptors are expressed in human medulloblastomas and ependymomas. (**A**) Human medulloblastoma microarray data from [36], categorized into the four molecular subgroups Group 3, Group 4, SHH, and WNT, were used to examine the expression of the indicated genes. (**B**) RNA-seq datasets from the two ependymoma subgroups, PFA (*n* = 46) and C11orf95 (*n* = 13), were analyzed for the genes indicated, and transcript abundance is shown as log2 transformed transcripts per million (TPM).

THC and CBD are known to elicit biological effects in mammalian cells via a range of diverse mechanisms. These include the binding of THC to CB_1_R and CB_2_R, in addition to THC and CBD binding to a range of other cellular proteins including adenosine receptor, TRP channels, peroxisome proliferator-activated receptors (PPARs), and other G-protein coupled receptors including GPR55 and GPR18 [33,34,35]. Online public databases were interrogated to determine the expression of these receptors in human medulloblastoma and ependymoma. Microarray RNA expression data from Cavalli et al [36]. comprising 763 medulloblastoma samples, was examined for expression of the genes encoding CB_1_R and CB_2_R: *CNR1* and *CNR2*. Both genes were expressed across all medulloblastoma subgroups (WNT (*n* = 70), SHH (*n* = 223), Group 3 (*n* = 144), and Group 4 (*n* = 326)), with CB_1_R expression highest in SHH subgroup tumors. The expression of other proteins known to elicit some effect of cannabinoids in human cells were also examined, including *PPARγ*, *GPR18*, *GPR55*, and the TRP channels *TRPV2*, *TRPV3*, *TRPV4*, *TRPA1,* and *TRPM8*. All genes were present in this data set (Figure 1A).

In order to assess expression of cannabinoid binding proteins in pediatric ependymoma, RNA sequencing (RNA-seq) was performed from a cohort of samples representing the PFA (*n* = 46) and C11orf95 (*n* = 13) molecular subgroups. *CNR1* was present, however transcripts for *CNR2* were few (Figure 1B). *TRPV2*, *TRPV4,* and *TRPA1* were expressed to similar levels across the two ependymoma subgroups. *PPARγ*, *GPR55,* and *TRPM8* transcripts were present to a higher level in PFA tumors, compared to C11orf95 tumors, while the inverse was observed for *TRPV3*. GPR18 expression was rarely observed. Overall, the expression of these genes was low, ranging from undetectable to approximately 128 transcripts per million (TPM). These RNA-seq results were validated in a microarray expression dataset from Pajtler et al. [11]. While the results were generally concordant, and *CNR1* expression was confirmed to be higher than *CNR2* expression, the differences between the PFA and C11orf95 subgroups were minimal in the microarray results (Figure S1A).

To determine if similar patterns of gene expression were observed in cultured pediatric brain cancer cells, as distinct from tumors, which are comprised of cancer cells as well as other cells such as normal brain, endothelial, and immune cells, we performed RNA-seq for three medulloblastoma cell lines (D283, D425 and PER547) and one ependymoma cell line (IC-1425EPN). These cell lines have been validated as accurate representations of *MYC*-amplified Group 3 medulloblastoma using DNA methylation array [37] and C11orf95 ependymoma using PCR [4]. *CNR1, PPARγ, GPR18,* and *TRPV2* were present in all cell lines but not *CNR2* or *GPR55* (Figure S1B). The other putative cannabinoid receptors were inconsistently expressed among the different cell lines.

### 2.2. THC and CBD Have Cytotoxic Activity in Pediatric Brain Cancer Cell Lines

**Figure 2:**
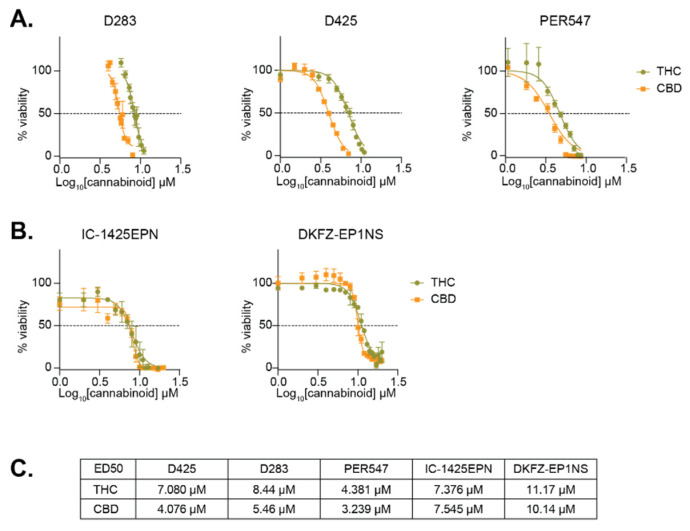
Cannabinoids reduce the viability of pediatric brain cancer cell lines. Dose response assays for THC and CBD were performed using the indicated (**A**) medulloblastoma and (**B**) ependymoma cell lines. Mean viability ± SEM were calculated from 3–5 independent experiments. The ED50 concentrations are summarized in (**C**).

Given the expression of *CNR1* in medulloblastoma and ependymoma samples and cell lines, along with other putative cannabinoid receptors, we investigated the effects of THC and CBD on the survival of these cells in vitro. Using three medulloblastoma cell lines (D283, D425, and PER547) and two ependymoma cell lines (IC-1425EPN and DKFZ-EP1NS), drug sensitivity assays indicated that both THC and CBD reduced cell viability in a dose dependent manner (Figure 2A,B). The effective dose that inhibits 50% of cells (ED50) was observed to be in the low micromolar range, with medulloblastoma cells appearing more sensitive to these compounds compared to ependymoma cells (Figure 2C). Interestingly, the ependymoma cells were equally sensitive to both THC and CBD, while medulloblastoma cells were consistently more sensitive to CBD than THC.

### 2.3. THC and CBD Mediated-Cytotoxicity May Be Mediated via the Production of Reactive Oxygen Species

**Figure 3:**
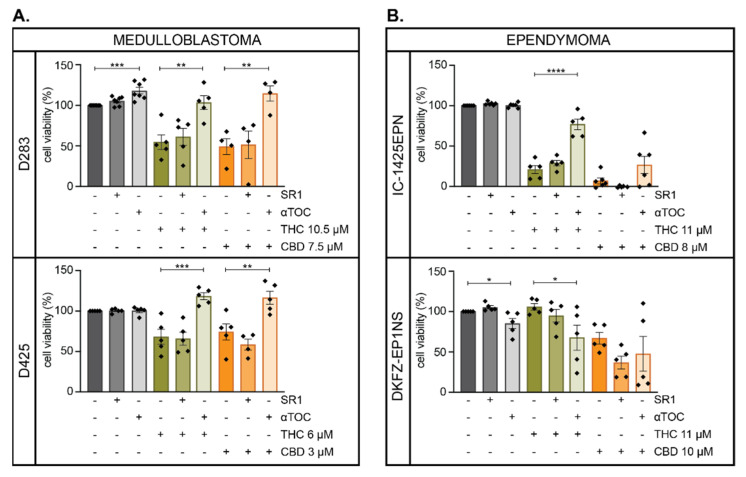
α-Tocopherol protects pediatric brain cancer cells from cannabinoid-induced cytotoxicity. (**A**) D283 or D425 medulloblastoma cells or (**B**) IC-1425EPN or DKFZ-EP1NS ependymoma cells, were treated with DMSO (grey), THC (green), or CBD (orange) at the concentrations shown in the absence (-) or presence (+) of the CB_1_R-selective antagonist SR141716 (SR1, 2 µM) or the antioxidant α-tocopherol (αTOC, 10 µM). The mean percentage (±SEM) of viable cells compared to DMSO controls was assessed after 72 h, where each dot represents the mean of triplicate wells from 4 or 5 independent experiments. Significant effects of SR1 and αTOC were determined using an ordinary one-way ANOVA with Bonferroni’s multiple comparisons test compared to DMSO, THC, or CBD only controls (*, *p* < 0.033; **, *p* < 0.0021; ***, *p* < 0.0002; ****, *p* < 0.0001).

To determine if THC was exerting effects on medulloblastoma and ependymoma cells via the activation of CB_1_R, cells were pre-incubated with the CB_1_R-selective antagonist SR141716 (SR1) followed by incubation with a sub-effective dose of THC, and cell viability was assessed using alamar blue assay after 72 h. As expected, THC reduced the viability of the cells; however, this was not prevented by the addition of SR1 (Figure 3). Although CBD is known to not act via CB_1_R, we also investigated the effects of SR1 on CBD-induced cytotoxicity, which confirmed that SR1 did not impact the activity of CBD. Another known mechanism of action for THC and CBD on cancer cells is the production of reactive oxygen species (ROS) [38,39]. To investigate if THC or CBD-induced cytotoxicity was mediated via ROS, cells were pre-incubated with the antioxidant α-tocopherol (αTOC). Remarkably, this resulted in almost a complete reversal of the effects of THC or CBD in the two medulloblastoma cell lines tested (Figure 3). αTOC also protected IC-1425EPN cells from THC; however, in the second ependymoma cell line, αTOC had negative effects on cell viability when used as a single agent, or in combination with THC. The cytotoxic effect of CBD in ependymoma cells were also not prevented by αTOC. Thus, THC and CBD appear to induce medulloblastoma cell death via the production of ROS and not via CB_1_R; however, the mechanisms of action in ependymoma cells are less straightforward.

### 2.4. CBD Induces Autophagy and Apoptosis in Pediatric Brain Cancer Cells Potentially via MAPK or mTOR Signaling

**Figure 4:**
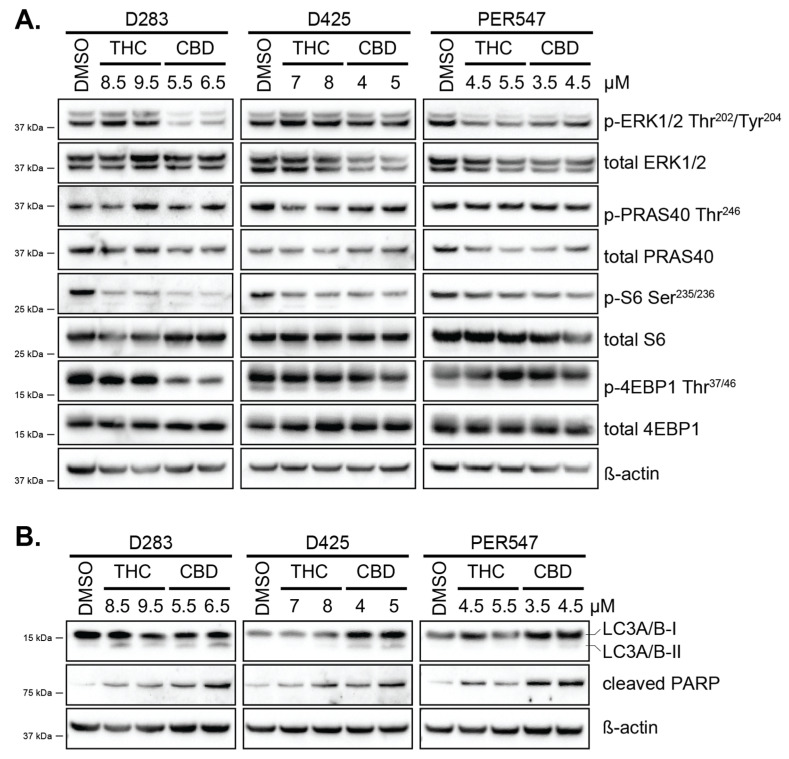
Cannabinoids inhibit intracellular signaling pathways and induce autophagy and apoptosis. (**A**) D283, D425, and PER547 medulloblastoma cells were treated with DMSO (0.1%), THC, or CBD at the concentrations shown and protein collected 48 h later. Immunoblot analysis was performed using the antibodies indicated. The locations of protein size markers are shown. (**B**) The induction of autophagy and apoptosis was investigated in the medulloblastoma cell lines shown via the detection of LC3A/B I and II and cleaved PARP, respectively, after 24 h drug exposure. Blots in (**A**,**B**) are representative of 2 or 3 independent experiments. Quantification of all immunoblotting data was performed using Image J (Figure S2).

The mechanisms by which THC and CBD act in pediatric brain cancer cells were further investigated by the analysis of intracellular signaling pathways via immunoblot. In adult glioblastoma cell lines and other cancer cell lines, the activity of phytocannabinoids has been shown to be mediated in part by the modulation of MAPK and/or AKT pathway activity [40,41]. The medulloblastoma cell lines D283, D425, and PER547 were incubated with DMSO, THC, or CBD, and protein lysates were harvested after 48 h. Immunoblotting was employed to detect changes in protein phosphorylation indicative of MAPK and AKT/mTOR signaling. Phosphorylation of ERK1/2 was reduced in the presence of CBD in D283 medulloblastoma cells (Figure 4), although due to experimental variability, this was not statistically significantly different to controls (Figure S2). In contrast, a significant increase in ERK1/2 phosphorylation was observed in a second medulloblastoma cell line (D425), while THC or CBD had no influence on ERK1/2 activity in a third cell line (PER547) (Figure 4 and Figure S2).

AKT phosphorylation in all medulloblastoma cells was low; therefore, phosphorylation of the AKT target PRAS40 [42] was used as a surrogate marker of AKT activity. No effects of THC or CBD on PRAS40 phosphorylation were observed in any of the medulloblastoma cell lines. In contrast, when we investigated further downstream in the pathway, differences in S6 and 4EBP1 phosphorylation were observed. S6 phosphorylation was consistently reduced by THC in two out of the three cell lines tested (Figure 4), although the reduction did not achieve statistical significance (Figure S2). CBD significantly reduced S6 and 4EBP1 phosphorylation in D283 medulloblastoma cells, but this was not observed in the other two cell lines. These data indicate that THC and CBD appear to inhibit both MAPK and AKT pathways in certain circumstances; however, the results are subtle, and intraexperimental variability was observed, suggesting that these effects are mild in medulloblastoma cells.

These modest results suggest that cannabinoids may be acting via additional intracellular mechanisms to reduce the viability of pediatric brain cancer cells. Cannabinoids have been shown to induce cancer cell death via the induction of autophagy [27,29,43]. Lysates from cells treated with THC or CBD for 24 h were investigated for the induction of LC3A/B-II as an indicator of autophagy. A small but statistically significant increase in LC3-II levels was observed following cannabinoid exposure in two out of three medulloblastoma cell lines, and this correlated with an increase in PARP cleavage (Figure 4B and Figure S2). Altogether, immunoblotting has revealed that THC and CBD can induce a small but significant amount of autophagy in medulloblastoma and ependymoma cells, possibly through the inhibition of MAPK and AKT signaling pathways, leading to the induction of apoptosis.

### 2.5. THC, CBD, or the Combination of Both Does not Impact Survival of Mice with Pediatric Brain Cancer

**Figure 5:**
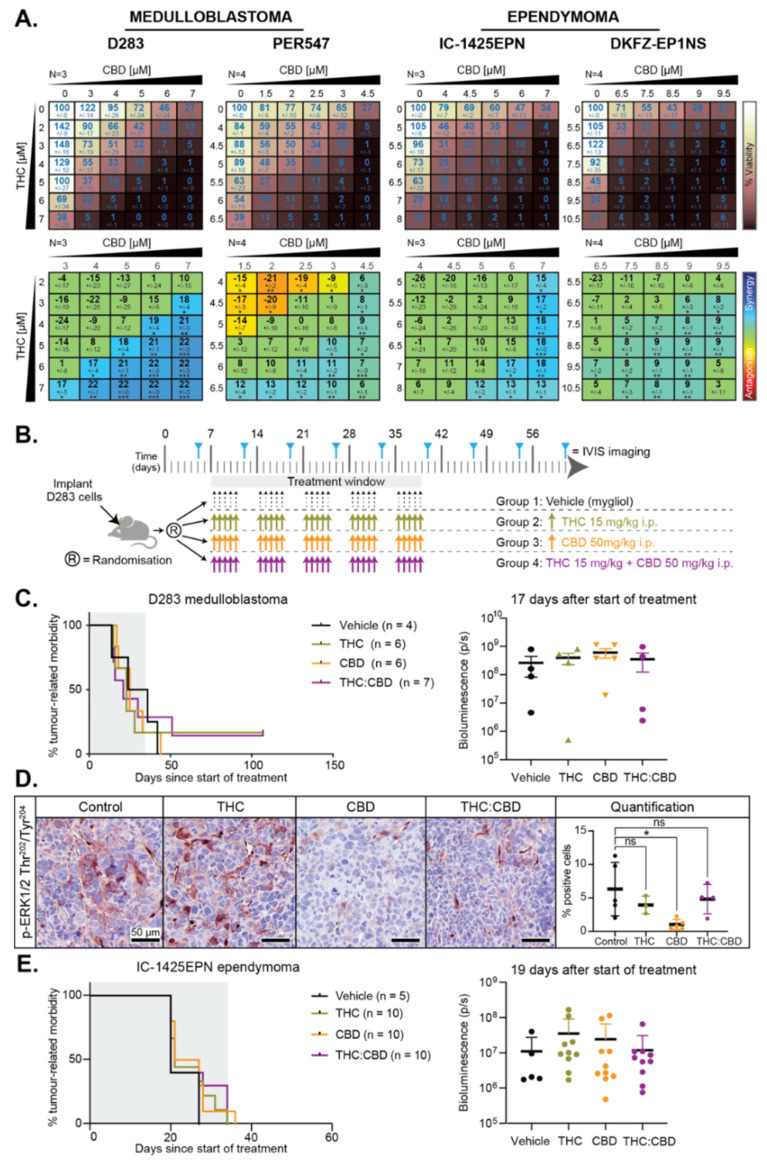
Cannabinoids do not improve survival in mouse models of medulloblastoma or ependymoma. (**A**) Medulloblastoma (D283 and PER547) or ependymoma (IC-1425EPN or DKFZ-EP1NS) cells were treated with the indicated concentrations of CBD (*x* axis) and THC (*y* axis). After 72 h, viability was normalized to DMSO-treated wells on each plate (top, indicated by the white/brown heat map). Drug interactions are presented as a matrix view of mean ± SD synergy/antagonism scores calculated using the Loewe Additivity model (bottom, rainbow heat map). The number of independent experiments (N) used in the analyses are shown, and statistical comparisons were performed as described previously [45] (*, *p* < 0.05; **, *p* < 0.01). (**B**) Preclinical treatment protocol for data shown in (**C**,**D**). (**C**) Survival curves for mice with D283 medulloblastoma xenografts treated as in B, with numbers of animals per group indicated (*n*). Bioluminescence measurements from the animals 17 days after treatment initiation are shown. (**D**) Representative immunohistochemistry images for phosphorylated ERK1/2 Thr^202^/Tyr^204^ from D283 medulloblastoma xenografts harvested 24 h after administration of vehicle (control), THC, CBD or combined THC:CBD. The mean percentage (±SEM) of positively-staining medulloblastoma cells was quantified from four independent fields of view per tumor from 3–5 mice per treatment group. Scale bar represents 50 µm. Staining intensity between groups was compared using one-way ANOVA with Bonferroni’s correction for multiple comparisons (*, *p* < 0.05). (**E**) Survival curves of mice with orthotopic IC-1425EPN ependymoma xenografts treated as in B except using 10 mg/kg THC and 30 mg/kg CBD. Number of animals per group is indicated (*n*). Bioluminescence measurements from the animals 19 days after the start of treatment are shown.

Several studies have shown that the effects of THC and CBD on cancer cells are enhanced when both cannabinoids are administered concurrently [24]. Drug interaction assays using two medulloblastoma and two ependymoma cell lines were performed to investigate if THC and CBD synergized to induce cytotoxicity in vitro. In these experiments, increasing concentrations of each drug are applied to cells as single agents or in combination. Mathematical models are then applied to determine if the observed experimental effect of using both drugs together is greater or less than what would be predicted based on the effects of each drug alone. Following drug exposure, cell viability was measured, and results were analyzed using the Loewe Additivity model [44] with Combenefit software [45]. In medulloblastoma cells, a significant synergistic interaction was observed in D283 cells, and mild but significant synergy was observed in PER547 cells, albeit with some antagonism detected at lower concentrations (Figure 5A). Mild synergy between THC and CBD was also observed in both ependymoma cell lines (Figure 5A).

To validate these results in vivo, mice were intracranially implanted with D283 medulloblastoma cells. Following tumor establishment, mice were treated as shown in Figure 5B for five consecutive weeks and euthanized upon the development of tumor-related morbidity. Median survival for mice treated with vehicle (Mygliol 812) was 30 days following the initiation of treatment. Mice treated with THC, CBD, or the combination of THC and CBD had median survival of 23, 25, and 21 days, respectively (Figure 5C). There were no statistically significant differences between any group. The medulloblastoma cells had been modified using retrovirus to express luciferase. Therefore, to confirm that tumor growth rates were not impacted by treatment, bioluminescence (photons per second per centimeter squared per steradian, abbreviated as p/s) was measured weekly as a surrogate measure of tumor growth. No difference in tumor burden across the treatment groups was observed (Figure 5C). The effects of THC or THC:CBD combination therapy were investigated in a second xenograft model of medulloblastoma induced by the orthotopic implantation of D425 medulloblastoma cells. Consistent with the D283 model, no change in survival was observed with THC or THC:CBD combination therapy (Figure S3).

It is well established that THC and CBD can cross the blood–brain barrier as indicated by the psychoactive effects of THC and anti-seizure effects of CBD; however, we wanted to confirm that the cannabinoids were penetrating medulloblastoma xenografts. As shown above, ERK1/2 phosphorylation was inhibited by CBD in D283 cells. To confirm that CBD was indeed present within D283 tumors, we performed immunohistochemistry on medulloblastoma xenografts within 24 h of treatment. Inhibition of ERK1/2 phosphorylation was observed in vivo following CBD but not THC treatment (Figure 5D), consistent with our in vitro data and confirming that CBD was having some effect on the tumor cells in vivo, even though this did not lead to any change in survival outcome.

To comprehensively assess the effects of THC and CBD in pediatric brain cancer, we also examined the effects of cannabinoid treatment in a model of ependymoma. IC-1425EPN cells were implanted intracranially, and bioluminescence was monitored until mice achieved a signal of approximately 10^6^ p/s prior to the initiation of treatment as illustrated in Figure 5B, except with a slightly reduced dose of drug. Following the initiation of treatment, approximately 30 days after implantation, median survival of mice with IC-1425EPN xenografts treated with vehicle was 20 days, 24 days for mice treated with THC, 21 days with CBD treatment, and 20 days for mice treated with the THC:CBD combination (Figure 5E). In summary, cannabinoid treatment neither improves nor reduces survival in mouse models of medulloblastoma or ependymoma.

### 2.6. Cannabinoids Alter the Cytotoxic Effects of the Conventional Medulloblastoma Chemotherapeutic Cyclophosphamide In Vitro

**Figure 6:**
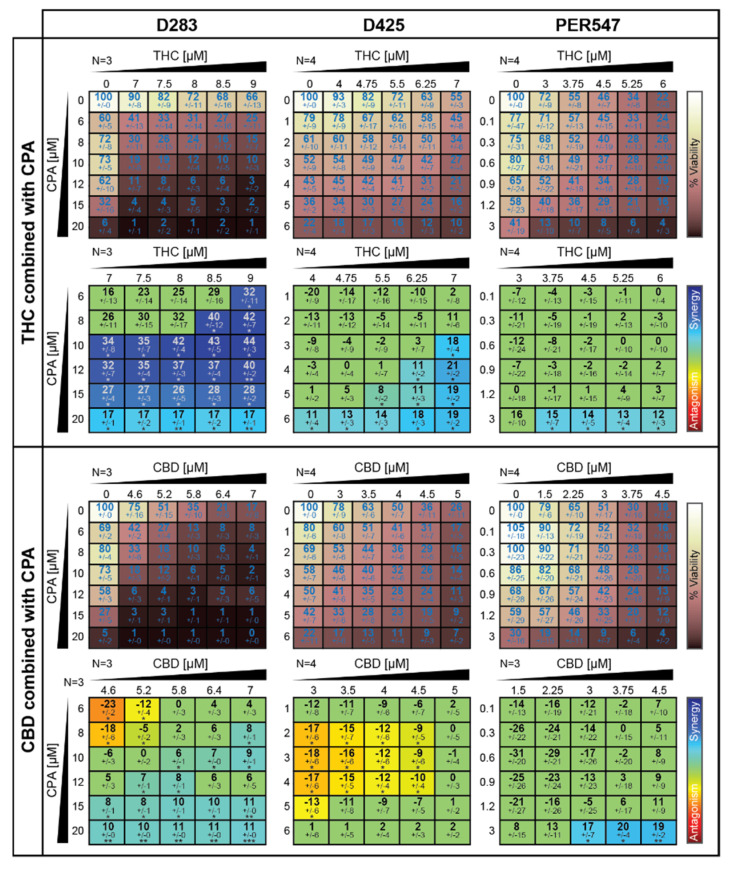
Cannabinoids alter the efficacy of cyclophosphamide on medulloblastoma cells in vitro. D283, D425, or PER547 medulloblastoma cells were treated with the indicated concentrations (along the *x* axis) of THC (upper panel) or CBD (lower panel) in combination with increasing concentrations of CPA along the *y* axis. Viability (indicated by the white/brown heat map) after 72 h was normalized to DMSO-treated wells on each plate. Drug interactions are presented as a matrix view of mean ± SD synergy/antagonism scores calculated using the Loewe Additivity model (rainbow heat map). The number of independent experiments (N) used in the analysis is shown, and statistical comparisons were performed as described in [45] (*, *p* < 0.05; **, *p* < 0.01; ***, *p* < 0.001).

Although cannabinoids appear to have minimal effects on the survival of pediatric brain cancer mouse models, there have been several published reports that suggest cannabinoids modulate both standard chemotherapy and radiation therapy in laboratory models of the adult brain cancer glioblastoma [24,25,26]. To determine if a similar role for cannabinoids exists in medulloblastoma, we investigated the effects of THC and CBD on medulloblastoma cells in combination with one of the major chemotherapeutics used in clinical treatment: cyclophosphamide (CPA) [46,47]. CPA is a pro-drug, therefore for in vitro experiments, the activated form of the drug, 4-hydroperoxycyclophosphamide (4HPC), was used. Drug interaction assays were again employed to examine if THC or CBD either synergized or antagonized the effects of CPA on three different medulloblastoma cell lines. Significant synergism was observed between THC and CPA, suggesting that THC is able to enhance the cytotoxic effects of CPA in medulloblastoma cells (Figure 6). In contrast, CBD appeared to have mixed effects with synergy observed at higher concentrations of both drugs, but antagonism was also observed in two out of three cell lines, suggesting that CBD might interfere with the effects of CPA (Figure 6).

### 2.7. Cannabinoids Induce Cell Cycle Arrest and Enhance CPA-Induced Apoptosis

**Figure 7:**
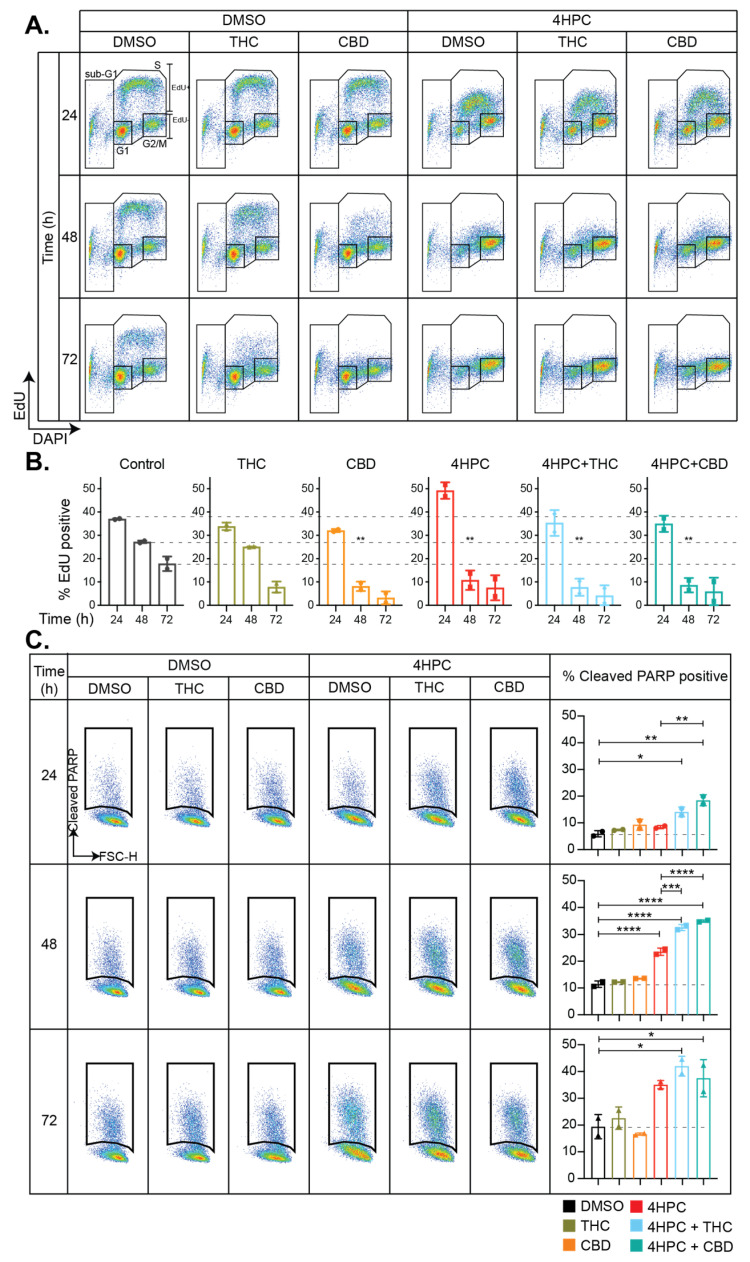
Cannabinoids influence cell cycle progression of medulloblastoma cells and enhance CPA-induced apoptosis. D283 medulloblastoma cells were treated with DMSO, THC, or CBD in the presence of DMSO or activated CPA (4HPC), and cell cycle distribution was assessed using flow cytometry. (**A**) Representative dot plots for EdU and DAPI are shown for the drug treatments and time points indicated. (**B**) Cells undergoing active DNA synthesis (marked with EdU) from two independent experiments were quantified and shown are mean ± SD. The dotted lines indicate control values for ease of comparison. (**C**) Apoptotic cells were determined using flow cytometry for cleaved PARP. Shown are representative dot plots and quantified data from two independent experiments. In B-C, the effects of THC and CBD were determined by comparing single-drug treated samples with DMSO controls, or combination treated samples with 4HPC alone. Statistical significance was established using one-way ANOVA with Bonferroni’s correction for multiple comparisons (*, *p* < 0.033; **, *p* < 0.0021; ***, *p* < 0.0002; ****, *p* < 0.0001).

CPA is an alkylating agent that causes DNA crosslinking, leading to cell cycle arrest and the subsequent induction of either DNA repair or apoptosis [48]. We used flow cytometry to examine the effects of CPA on cell cycle phase distribution and the induction of apoptosis, and to investigate if THC or CBD modulated DNA-damage induced cell cycle changes in D283 medulloblastoma cells. Cells were treated with DMSO, THC, or CBD in the presence or absence of 4HPC and cell cycle distribution was examined 24, 48, and 72 h post-drug exposure by staining the cells for EdU to identify cells in S phase, phospho-histone H3 for cells in mitosis, and DAPI for DNA content (Figure 7A,B). The gating strategy used to identify cells in each part of the cell cycle is shown in Figure S4. Consistent with the effects of THC and CBD on medulloblastoma cell viability in vitro, the cannabinoids induced cell cycle arrest when used at sub-ED50 doses, with CBD significantly decreasing the number of cells undergoing active DNA synthesis after 48 h exposure (Figure 7A,B). As expected, CPA also induced cell cycle arrest, reducing the proportion of cells traversing through S phase. When CPA was used in combination with THC or CBD, no significant differences in DNA synthesis were observed when compared to cells exposed to CPA alone. Of note, CPA induced a rapid reduction of mitotic cells, but mitosis returned to control levels by 48 h. In contrast, when cells were co-treated with THC and CPA or CBD and CPA, the re-initiation of mitosis was significantly delayed (Figure S5, 48 h).

The effects of drug exposure on the induction of medulloblastoma cell apoptosis was examined by the detection of cleaved PARP. Despite the presence of cleaved PARP in immunoblots within 48 h of exposure to THC or CBD (Figure 4), there was no significant increase in apoptosis observed by flow cytometry when the cannabinoids were used alone. CPA increased the proportion of apoptotic D283 cells as expected, but notably this was not observed until 48 h post-drug exposure. However, when THC or CBD were combined with CPA, a significant induction of apoptosis was observed at 24 h. This suggests that cannabinoids can enhance CPA-induced apoptosis of medulloblastoma cells in vitro.

### 2.8. Cannabinoids Do Not Enhance the Efficacy of CPA In Vivo and Do Not Improve Animal Survival

**Figure 8:**
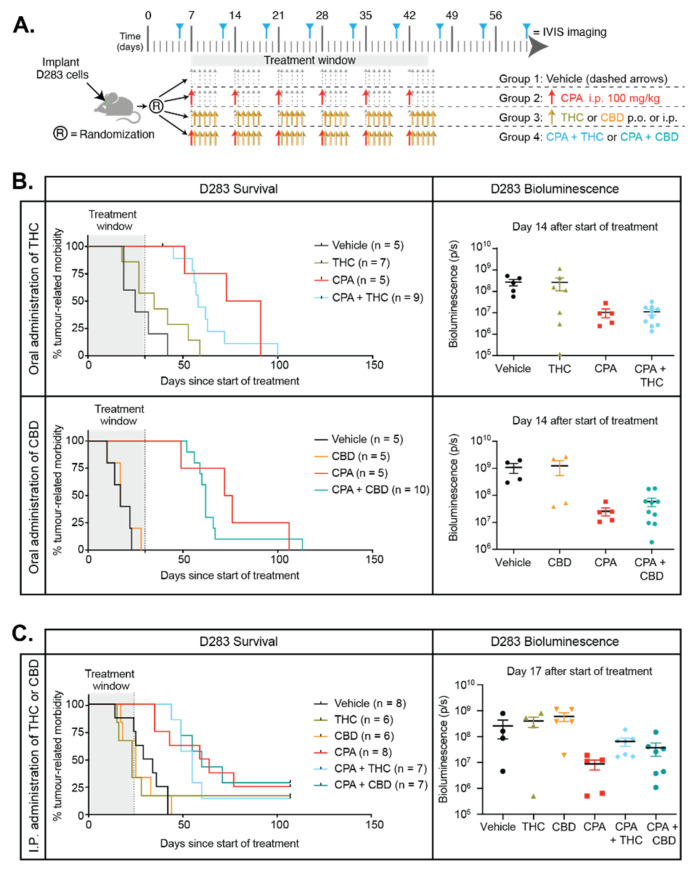
Cannabinoids do not enhance the ability of CPA to extend survival of mice with medulloblastoma. (**A**) Preclinical treatment protocol for mice with orthotopic D283 medulloblastoma xenografts for data shown in B,C. (**B**) The effects of oral administration of THC (15 mg/kg, top panel)) or CBD (50 mg/kg, bottom panel) administered alone, or in combination with CPA (100 mg/kg i.p.), were determined using Kaplan–Meier survival analyses (left panels). Bioluminescence measurements from the animals 14 days after the start of treatment are shown. (**C**) Survival curves from mice with D283 medulloblastoma treated i.p. with vehicle (Miglyol 812, black), THC (15 mg/kg, green), CBD (50 mg/kg, orange), CPA (100 mg/kg, red), the combination of THC and CPA (blue), or the combination of CBD and CPA (teal) are shown. Bioluminescence measurements from the animals 17 days after the start of treatment are shown on the right. For all survival curves, the numbers of mice per group are indicated (*n*) and the difference between curves was evaluated using log-rank tests.

The synergistic interactions between cannabinoids and CPA, together with the enhanced apoptosis of medulloblastoma cells exposed to combinations of THC:CPA or CBD:CPA, encouraged us to hypothesize that cannabinoids may improve the effects of chemotherapy in mouse models of medulloblastoma. We investigated this in mice with orthotopic D283 medulloblastoma xenografts. Mice were treated as shown in Figure 8A. Tumor growth was assessed using bioluminescence imaging, and the effects of treatment were compared using Kaplan–Meier analyses. Cannabinoids were initially administered via oral gavage (*per os*, p.o.), as this would be the preferred route of administration for children. Orally administered THC or CBD did not change the median survival of mice with medulloblastoma (Figure 8B). These experiments were performed as two independent cohorts. In the experiment combining THC with CPA, median survival for vehicle controls was 25 days, and for THC it was 35 days, although these are not significantly different according to log-rank tests. As expected, CPA is an effective drug against this disease, resulting in significantly extended median survival of 82 days, while when THC was administered with CPA, median survival was 58 days, and not significantly different to CPA alone. In the experiment testing CBD in combination with CPA, median survival of both the vehicle controls and CBD treated mice was 17 days after the start of treatment. CPA extended survival to 74 days, and for mice treated with CBD and CPA in combination, median survival was 62 days. Tumor burden in both experiments was examined using bioluminescence imaging, and no significant differences were observed between vehicle and THC-treated mice, vehicle and CBD-treated mice, CPA and THC:CPA combination treated mice, or CPA and CBD:CPA combination treated mice (Figure 8B, right).

These data suggest that the enhanced medulloblastoma cell apoptosis observed in vitro when cannabinoids were combined with CPA does not translate to improved survival in vivo. Given that cannabinoids rarely reach 20% bioavailability when delivered orally [49], we wanted to test an alternative route of administration to ensure that our observations were as robust as possible. Therefore, an additional cohort of mice with D283 medulloblastoma were treated with THC:CPA or CBD:CPA combinations where the cannabinoids were administered intraperitoneally (i.p.) (Figure 8C). Changing the route of administration did not alter the effects of treatment. When administered i.p., THC or CBD did not alter the median survival of mice compared to vehicle treated controls. Furthermore, combining THC or CBD with CPA did not improve median survival significantly compared to CPA alone. These data conclusively demonstrate that the phytocannabinoids THC and CBD do not appear to increase the survival of mice with medulloblastoma; however, importantly, our results demonstrate that cannabinoids do not adversely affect or interfere with the actions of CPA in vivo.

**Figure 9:**
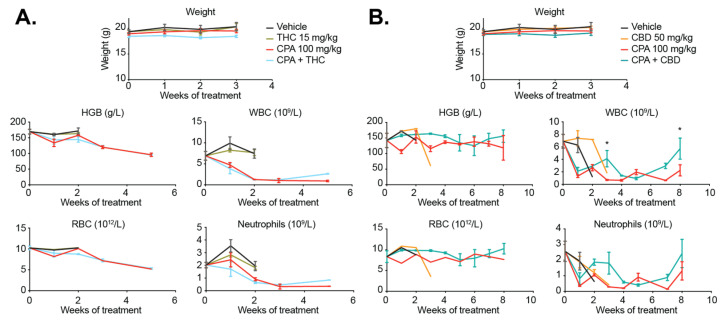
Cannabinoids are well tolerated in mice and may protect against CPA-induced toxicity. Body weight and blood parameters were analyzed from mice treated with (**A**) vehicle (black), CPA (100 mg/kg i.p., red), THC (15 mg/kg p.o., green), or the combination of CPA and THC (blue), or (**B**) CBD (50 mg/kg p.o., orange) or the combination of CPA and CBD (teal). The treatment schedule is described in Figure 8A, and blood was harvested weekly from mice in the experiments shown in Figure 8B. Blood parameters assessed were hemoglobin (HGB), red blood cells (RBC), white blood cells (WBC), and neutrophils. (*, *p* < 0.05).

In pilot experiments, we observed that if cannabinoids were administered at the same time as CPA, mice exhibited weight loss requiring euthanasia. Both CPA and cannabinoids are processed by similar enzymes in the liver [50,51], which may alter drug excretion. Therefore, in subsequent experiments, cannabinoids were administered 3 h after CPA, and animals were closely monitored for signs of treatment-related toxicity. Body weight measurements, along with the assessment of hemoglobin levels, red and white blood cell counts, and neutrophil counts, show that cannabinoids are well tolerated in mice at the dosages tested over an extended treatment window (Figure 9). As is well established, CPA causes hematopoietic toxicity [52], which is replicated in our mice by a reduction in white blood cells and neutrophils. Although THC was unable to rescue these effects in mice, mice concurrently treated with CBD and CPA appeared to have improved white cell counts compared to mice treated with CPA alone. These data suggest that CBD may mildly protect against some CPA-induced toxicity.

## 3. Discussion

Tumors arising in the CNS are the most common solid cancers of childhood and the major cause of childhood cancer deaths [53]. This research study has focused on two aggressive pediatric brain cancers that affect young children particularly—medulloblastoma and ependymoma. Despite multimodal treatment protocols including surgery, radiotherapy, and chemotherapy, survival rates for patients with high-risk disease have failed to improve significantly for several decades, and recurrences are common. Extensive molecular characterization of both cancers [3,11] has not led to successful translation of many novel therapies to the clinic, even with focused efforts to develop drugs that target specific mutations in these diseases. In addition, survivors frequently encounter significant long-term sequelae including developmental defects, psychosocial deficits, and secondary tumors [54]. Thus, additional improvements are required for patients with aggressive disease, and new treatment approaches must focus on innovative ways to reduce the long-term toxicity of therapy [55].

Recent research has demonstrated that cannabinoids, including the phytocannabinoids THC and CBD but also synthetic cannabinoids, exhibit anti-tumor properties in different adult cancer types, including breast cancer, melanoma, pancreatic cancer, lymphoma, and glioblastoma [23,56]. The robustness of these studies has resulted in the implementation of new clinical trials, specifically in adult glioblastoma, that are investigating the cannabis-based medicine Sativex (GW Pharmaceuticals) in combination with temozolomide (ClinicalTrials.gov ID: NCT01812603 and NCT01812616). In addition, it has been established that cannabinoids, particularly CBD, exhibit minimal toxicity, may reduce some brain tumor-related symptoms such as seizures, and are well tolerated by patients [57]. Despite these encouraging data, and the known ability of cannabinoids to penetrate the blood–brain barrier, there is no existing pre-clinical data on the effect of these agents in pediatric brain cancer.

In order to investigate the role of THC and CBD in medulloblastoma and ependymoma, we took advantage of existing in vitro models of each disease [4,58,59,60,61]. Specifically, these models represent *MYC*-amplified Group 3 medulloblastoma and *C11orf95* fusion-positive ependymoma. In addition, we used multiple cell lines of these two molecular subtypes to confirm the reproducibility of our findings across multiple models. Encouragingly, we observed expression of both canonical and non-canonical cannabinoid receptors within medulloblastoma and ependymoma samples, as well as the cell lines. Given this was the first study of these compounds in pediatric brain cancer, we chose to investigate purified THC and CBD, rather than utilize plant extracts, which contain a complex mixture of many additional compounds with potential therapeutic properties [62]. While it has been demonstrated that whole-plant cannabis preparations may elicit better therapeutic effects [63], we aimed to first determine if THC and CBD had any specific effect on pediatric brain cancer cells. Our data demonstrate that both compounds influence medulloblastoma and ependymoma cells, although in different ways.

Consistently, both CBD and THC reduced the viability of these cell lines, although the medulloblastoma cell lines were more sensitive than ependymoma cells. This was particularly encouraging given that the cell lines used represent subgroups of each disease with poor prognosis. Surprisingly, the effects of THC did not appear to be mediated via CB_1_R, but instead cannabinoid-induced toxicity could be prevented by α-tocopherol in three out of the four cell lines tested, suggesting that these drugs induce ROS within brain cancer cells. Similar mechanisms of action have been demonstrated in glioblastoma cells where ROS induction was linked to increased apoptosis [38]. However, neither THC nor CBD induced significant apoptosis in medulloblastoma cells when assessed by flow cytometry. This may be due to the concentrations of THC and CBD used, which were below the concentrations required to completely inhibit viability. The effect of α-tocopherol in ependymoma cells was less clear with it appearing to have a negative effect on DKFZ-EP1NS cell viability.

Given the demonstrated roles for THC and CBD in the modulation of intracellular signaling pathways, we investigated their effects primarily in medulloblastoma cells. In general, an overall decrease in signaling pathway activity was observed; however, these changes were rarely statistically significant when multiple experiments were quantified. In the case of ERK1/2 activity, disparate effects of CBD were observed with one cell line having reduced activity, one with increase phosphorylation, and the other with no change. The variability in response might be due to differences in the underlying genetics of each cell line used; however, given that we have selected three medulloblastoma cell lines with the same molecular classification, these differences have been experimentally minimized. Moreover, it has been shown that cannabinoids have a biphasic effect on ERK1/2 phosphorylation [40]; thus, it is feasible that timing in response to THC or CBD may differ across cell lines. Both THC and CBD appeared to increase autophagy in medulloblastoma cells, similar to what has been reported in cultured glioblastoma cells [29]. To date, molecular markers that predict how cancer cells respond to cannabinoids have not been reported but would be useful to distinguish cancers that might be inhibited by these agents.

Studies have shown that combining different cannabinoids, especially THC and CBD, can potentiate the effect of each cannabinoid. It was encouraging that THC and CBD appeared to synergistically reduce medulloblastoma and ependymoma cell viability in vitro; however, when tested in vivo, no survival benefit of either drug, or the combination of THC and CBD, was observed. This contrasts with studies in other brain cancers, where the combination of THC and CBD was superior to the use of each drug as a single agent in vivo [24]. One major difference is that Torres et al. utilized ectopic models of glioblastoma [24]. Another study that used an intracranial model of glioblastoma showed that the combination of THC with CBD did not improve animal survival, supporting our data, although they did see a benefit of cannabinoids when they were combined with temozolomide [64]. This approach of combining multiple different drugs has underpinned much of our past success in the treatment of pediatric cancer; thus, we tested the combination of THC and CBD with the medulloblastoma drug CPA. THC and CBD appeared to exhibit in vitro synergy with CPA; however, these effects were also not replicated in vivo. It is conceivable that the differences between the in vitro and in vivo effects of cannabinoids observed in our and other’s studies are due to their bioavailability and pharmacokinetics. It has been reported that the bioavailability of cannabinoids after oral administration can vary considerably, because they need to be absorbed in the intestine, then metabolized in the liver, before incorporation to the blood stream, thus affecting the cannabinoid concentrations reaching the tumor and consequently impacting any anti-tumor effect. Different studies have compared the pharmacodynamics and pharmacokinetics of cannabinoids in tumor-free mice and rats after different routes of administration, showing that the concentration of CBD in plasma or in brain after oral administration is much lower than intraperitoneal or intravenous administration [49,65]. To address the potential poor intra-tumoral drug concentrations that may result from oral administration of cannabinoids, we tested an alternative route of administration of these compounds, but this also did not improve overall survival in the models tested. A limitation of our study is that we did not measure intratumoral cannabinoid concentrations using mass spectrometry, although the inhibition of ERK1/2 phosphorylation we observed within medulloblastomas indicated that sufficient CBD is present within medulloblastoma xenografts to inhibit signaling; however, we did not have a molecular marker demonstrating effects of THC in these cells to validate intra-tumoral drug penetration.

Previously, we reported drug interaction studies between the pan-ErbB inhibitor dacomitinib with CPA using these same medulloblastoma cell lines and found additive or antagonistic in vitro drug interactions were in fact antagonistic in vivo [66]. Antagonism was not observed when THC or CBD were combined with CPA in vivo, which possibly indicates that although cannabinoids do not improve survival, they do not appear to inhibit the action of conventional chemotherapy. Our flow cytometry data indicated that CBD inhibited medulloblastoma proliferation. Since many anti-cancer chemotherapies, including CPA, require cells to be actively proliferating for cytotoxicity, this CBD-induced cytostasis may be the reason there was no improvement in survival when it was combined with CPA in vivo. A limitation of this study is that we only assessed one of the chemotherapies used in medulloblastoma treatment and have not investigated the influence that THC or CBD may have on other standard agents used in medulloblastoma or ependymoma, such as cisplatin, vincristine, etoposide, or radiation. It has been shown that phytocannabinoids have suppressive effects on immune cells [67]; however another limitation of our research is that we evaluated the effectiveness of THC and CBD using human brain cancer cells xenografted into immune-deficient mice. While preclinical studies that showed cannabinoids are effective against adult glioblastoma used similar immune-deficient models, it may be useful to determine if the presence of a functioning immune system might alter the effects of cannabinoids on medulloblastoma and ependymoma.

Although no direct anti-tumor effect was observed in our in vivo experiments, cannabinoids have been proven to have numerous other positive effects for cancer patients. For example, modulation of the endocannabinoid system has been shown to prevent cisplatin-induced neuropathic pain in preclinical models [68], suggesting cannabinoids may potentially improve patient quality of life; however, these data have not yet been validated in a clinical setting. Regardless of the potential positive effects of THC and CBD, the possibility that they may interact with other cancer treatments must be noted. THC and CBD are predominantly metabolised in the liver by cytochrome p450 family enzymes [51]. Specifically, they are primarily metabolised by CYP3A4 [50,51], which also metabolises several brain cancer chemotherapeutics including CPA and vincristine [69,70]. Therefore, although we did not observe additional toxicity in our mouse models when THC or CBD were combined with CPA, there is the potential that co-administration of cannabinoids with conventional cancer treatments could alter the bioavailability of chemotherapeutics, prolonging their cytotoxic effects in children.

In summary, we have comprehensively assessed the effects of THC and CBD in mouse models of medulloblastoma and ependymoma and investigated the intracellular effects of these compounds using multiple cell lines that represent two molecular subtypes with poor survival outcomes. While it could be argued that better efficacy may have been observed in models representing less aggressive disease subgroups, those tumors currently respond well to existing therapies, and our goal was to focus on identifying improved therapeutics for subgroups of these diseases most likely to relapse with current gold standard therapies. THC and CBD do not appear to elicit an in vivo survival benefit in mouse models of medulloblastoma or ependymoma. Given the lack of therapeutic efficacy in these cancer models and insufficient data demonstrating other benefits in children, future studies focusing on the potential for these compounds to improve quality of life are required to build more evidence for the use of these drugs in pediatric neuro-oncology.

## 4. Materials and Methods

### 4.1. Analysis of Human Medulloblastoma and Ependymoma Expression Data

Boxplots were generated using the R statistical environment to infer the levels of mRNA expression seen in human ependymoma and medulloblastoma samples. Two ependymoma datasets were analyzed: (1) GSE64415 [11] and (2) RNA-seq from formalin-fixed paraffin embedded (FFPE) tissue in UK ependymomas (Ritzmann et al. in preparation, data available upon reasonable request). One medulloblastoma dataset was analyzed (GSE85217 [36]). All samples were supported by DNA methylation-based subclassification [71]. For ependymoma, the predominant pediatric subgroups were included (EPN_PFA, EPN_C11orf95), and for medulloblastoma, the four main subgroups were analyzed (WNT, SHH, Group 3, Group 4). For the two publicly available datasets, the normalized data were downloaded from the Gene Expression Omnibus (GEO) and levels of expression plotted per subgroup. For the RNA-seq expression set, data was generated by 100bp paired-end RNA-seq on total RNA extracted from FFPE tissue following ribodepletion with Ribo-Zero. Libraries were sequenced on an Illumina HiSeq machine targeting 50 million reads per sample and aligned to the human genome (Hg19) and transcriptome using TopHat2 and summarized at gene level using FeatureCounts. DNA Methylation analysis for the RNA-seq dataset was performed as previously described [6]. Samples were normalized to log2 transcripts per million (log2TPM) for visualization.

### 4.2. Medulloblastoma and Ependymoma Cell Lines and Culture Conditions

D425 and D283 cells [58,59] were a gift from Darell Bigner of Duke University (Durham, NC, USA), and PER547 cells [60] were a gift from Ursula Kees of Telethon Kids Institute (Perth, Australia). The genetic identity of medulloblastoma cell lines was confirmed by STR analysis and sequencing. The IC-1425EPN cells were gifted by Xiao-Nan Li (Northwestern University, Chicago, IL, USA) [4], while the DKFZ-EP1NS cells were provided by Till Milde (German Cancer Research Center (DKFZ), Heidelberg, Germany) [61]. Cells were confirmed to be mycoplasma-free using a MycoAlert™ Mycoplasma Detection Kit (Lonza, Basel, Switzerland). Cells were transduced to express luciferase using the retroviral expression construct MSCV-ires-pacLuc2 (D283 and PER547) or lentiviral expression construct pCL20-MSCV-GFP-ires-Luc2 (D425). Constructs were generously supplied by Drs Suzanne Baker, Richard Williams, and Arthur Nienhuis, of St Jude Children’s Research Hospital (Memphis, TN, USA). Medulloblastoma cell lines were cultured in antibiotic-free media supplemented with Glutamax (Invitrogen, Carlsbad, CA, USA) at 37 °C in 5% CO_2_ as follows: D283: MEM-alpha (Gibco, Waltham, MA, USA) with 10% fetal bovine serum (FBS) (CellSera, Rutherford, Australia); PER547: RPMI (Gibco) with 1 mM sodium pyruvate (Invitrogen), non-essential amino acids (Invitrogen), 50 µM 2-mercaptoethanol (Sigma-Aldrich, St Louis, MO, USA), and 10% FBS; D425: modified IMEM (Gibco) with 1 M HEPES (Gibco) and 10% FBS. Ependymoma cell lines were cultured in media supplemented with Glutamax and antibiotics at 37 °C in 5% O_2_ and 5% CO_2_ as follows: IC-1425EPN (short term cultures of cells isolated from xenografts): DMEM:F12 (Gibco), 10% FBS, 1 µg/mL heparin (Sigma Aldrich, St Louis, MO, USA), 20 ng/mL epidermal growth factor (EGF), and 20 ng/mL basic fibroblast growth factor (FGF) (both from Peprotech, Rocky Hill, CT, USA); DKFZ-EP1NS: Neurobasal medium without vitamin A (Gibco), B27 with vitamin A (Gibco), N2 (Gibco), 20 ng/mL EGF, 20 ng/mL FGF, and 1 µg/mL heparin.

### 4.3. RNA Isolation and Transcriptome Profiling (RNA-Seq) from Cell Lines

D283, D425, PER547, or IC-1425EPN cells were cultured as described above, and total RNA was extracted using an AllPrep DNA/RNA mini kit (Qiagen, Hilden, Germany). RNA integrity was assessed by bioanalyzer (RIN 10). Total RNA was shipped to the Australian Genome Research Facility for library preparation and sequencing (Illumina NovaSeq 6000, 150bp paired-end (PE) reads). Between 12–59 million sequencing reads were generated per sample. The raw sequencing data are available from the European Genome-Phenome Archive (Accession number: EGAS00001004963).

### 4.4. Pre-Processing of RNA-Seq Data

FASTQC [72] was employed for pre-alignment quality control of raw sequence reads. Reads were aligned to the human reference genome (hg38) using HISAT2 [73] and summarized at the gene-level using featureCounts [74]. Post-alignment QC was carried out with SAMStat [75]. The proportion of mapped reads was 93% (range 92.3–5.0%). A gene was deemed expressed with a count ≥ 10 in at least one cell line and visualized showing counts per million (cpm), which normalizes each cell line with respect to sequencing depths.

### 4.5. Compounds

THC and CBD were purchased from THC Pharm GmbH (Frankfurt, Germany). THC was dissolved in ethanol and stored at −20 °C. Prior to use, ethanol was evaporated in a siliconized tube under a nitrogen stream. CBD was provided as a powder and stored at room temperature. The cannabinoids were either dissolved in DMSO (10 mM) (Sigma Aldrich) for in vitro use, or dissolved directly in Miglyol 812 (IOI Oleochemical GmbH, Hamburg, Germany) for in vivo administration either *per os* (p.o., oral gavage) or intraperitoneally (i.p.). Dosages are indicated in the text. CPA (Endoxan; Baxter Healthcare, Deerfield, MA, USA) was dissolved in saline and delivered i.p. as described in the text. For in vitro studies, the activated form of CPA was used, and 4HPC (Toronto Research Chemicals, Toronto, ON, Canada) was dissolved in DMSO and stored at −80 °C. The CB_1_R-selective antagonist SR141716A (SR1) was from MedChemExpress (Monmouth Junction, NJ, USA) and stored at 80 °C, and α-tocopherol (αTOC) was from Sigma-Aldrich. Both compounds were dissolved in DMSO for in vitro experiments.

### 4.6. Drug Sensitivity and Drug Interaction Assays

Cannabinoids are known to bind serum proteins; therefore, drug sensitivity was assessed in low serum conditions (1.5% FBS). Cells were plated (1500/well) into tissue culture treated 384-well plates (Corning, New York, NY, USA) using a Multidrop Combi (Thermo Scientific, Waltham, MA, USA). Medulloblastoma and ependymoma cells were incubated for 1 and 24 h, respectively, prior to the addition of drug. Drugs (either single drugs or drug combinations) were dispensed using an HP300 digital dispenser (Tecan, Mannedorf, Switzerland) with concentrations indicated in the text. Cells were treated for 72 h and incubated with alamar blue (2.5% methylene blue, 1 mM potassium hexacyanoferrate (III), 1 mM potassium hexacyanoferrate (II) trihydrate, and 0.6 mM resazurin (all from Sigma-Aldrich)) for the final 6 h of treatment. Resorufin fluorescence was detected using a SynergyMX plate reader (Biotek, Winooksi, VT, USA) with 570 nm excitation and 590 nm emission. Data were expressed as a percentage of DMSO-treated controls present on each plate. The ED50 was interpolated from a best-fit dose–response curve determined using Prism v8 (GraphPad Software, San Diego, CA, USA). Drug interactions were analyzed using Combenefit software (Cambridge University, Cambridge, UK) [45].

### 4.7. Cannabinoid Receptor Antagonist Assays

Cells were washed and resuspended in 1.5% FBS prior to plating (6000/well) into tissue culture-treated 96-well plates using a Multidrop Combi (Thermo Scientific, Waltham, MA, USA). Drugs were dispensed using an HP300 digital dispenser (Tecan) in a combination array matrix. Cells were treated with 2 µM of CB_1_R-selective antagonist SR1 or 10 µM of antioxidant αTOC for 1 h [41,76]. THC and CBD were then added (concentrations indicated in the text) for 72 h, and viability assessed using alamar blue as above. The reduction of resazurin to resofurin was measured via absorbance at 600 nm using a SynergyMX plate reader. Raw absorbance data was normalized to the absorbance measured in the corresponding control for each treatment and expressed as a percentage of control.

### 4.8. Protein Analysis by Western Transfer and Immunoblotting

PER547, D283, and D425 cells were washed and resuspended twice in medium containing 1.5% FBS and incubated for 3 h before being treated with the ED50 and ED80 doses of THC and CBD. Cells were lysed after 24 or 48 h with radioimmunoprecipitation assay (RIPA) buffer containing protease and phosphate inhibitors (Roche, Basel, Switzerland). Protein concentration was quantified using BCA assay (Pearce, Appleton, WI, USA) and 30 µg/lane separated using 4–12% NuPAGE Bis-Tris gels (Invitrogen) followed by transfer onto nitrocellulose membranes. Membranes were immunoblotted with specific primary antibodies, followed by horseradish peroxidase-conjugated secondary antibodies (1:5000) (GE Healthcare, Chicago, IL, USA). Relevant bands were detected using Supersignal West Dura (Pierce) or Clarity Western ECL (BioRad, Hercules, CA, USA) and images collected using a BioRad ChemiDoc. Primary antibodies used were PRAS40 (Cell Signaling Technologies (CST, Danvers, MA, USA) #2691, 1:1000), phosphorylated PRAS40 Thr^246^ (CST #2997, 1:1000), p42/44 ERK1/2 (CST #9102, 1:1000), phosphorylated ERK1/2 Thr^202^/Tyr^204^ (CST #9101, 1:1000), S6 ribosomal protein (CST #2217, 1:1000), phosphorylated S6 ribosomal protein Ser^235/236^ (CST #2211, 1:1000), 4EBP1 (CST #9452, 1:1000), phosphorylated 4EBP1 Thr^37/46^ (CST #2855, 1:1000), LC3A/B (CST #12741, 1:1000), cleaved PARP Asp^214^ (CST #5625, 1:1000), and β-actin (Sigma-Aldrich #A1978, 1:5000).

### 4.9. Flow Cytometry for Cell Cycle Distribution and Apoptosis

Cell cycle distribution was analyzed using EdU (added 45 min before harvest) to label cells in S phase and DAPI to label DNA content. D283 cells were treated with DMSO (0.1%), 7.5 μM THC or 5.5 μM CBD, in the presence of DMSO or 10 μM 4HPC. Time of harvest is indicated in the figures. Cells were stained using the Click-iT EdU AlexaFluor488 kit (Invitrogen). In addition, cells were stained with AlexaFluor647-conjugated cleaved PARP (CST #68975, 1:50) to identify apoptotic cells, and PE-conjugated phospho-histone H3 Ser10 (CST #5764, 1:50) to mark cells in mitosis. Samples were analyzed using an LSRFortessa X20 (BD, Franklin Lakes, NJ, USA) and results were visualized and quantified using FlowJo software. Data are pooled from two independent experiments and show the mean with standard deviation (SD).

### 4.10. Orthotopic Implant Models of Medulloblastoma and Ependymoma

Animal experiments were approved by the Animal Ethics Committee of the Telethon Kids Institute and performed in accordance with Australia’s Code for the Care and Use of Animals for Scientific Purposes. For survival studies, cells (100,000 per mouse) were suspended in Matrigel (Corning) and implanted into the right cerebellar hemisphere of 6–10-week-old female athymic mice (Balb/c nude) or NRG mice for medulloblastoma and ependymoma cells, respectively (Animal Resources Centre, Perth, Western Australia, Australia) using a Hamilton syringe as previously described [77]. Tumor size was monitored by bioluminescence using a LagoX Optical Imager (Spectral Instruments Imaging, Tucson, AZ, USA). Once tumors were established, mice were randomized into groups based on bioluminescence flux to obtain groups of mice with close to equal mean bioluminescence prior to treatment as indicated in the text. For D283, treatment commenced seven days after implantation. For IC-1425EPN, treatment commenced once the average radiance reached 10^6^ p/s. Median survival and Kaplan–Meier survival curve comparisons were calculated using GraphPad Prism (v8). An event was recorded when mice were euthanized due to intracranial tumor-related morbidity. Mice requiring euthanasia for non-tumor-related reasons (e.g., weight loss, infection, and physical trauma) were censored. Whole blood was collected weekly, treated with ethylenediaminetetraacetic acid, and parameters measured using a BC-5000VET Hematology Analyzer (MindRay, Shenzhen, China).

### 4.11. Immunohistochemistry

Mouse brains were embedded in paraffin after fixation in 4% paraformaldehyde in PBS overnight at 4 °C. Tissue sections (5 µm) were processed in a citrate buffer for antigen retrieval before immunostaining with phosphorylated ERK1/2 (pERK1/2) Thr202/Tyr204 (CST #9101, 1:200). An Elite ABC kit with NovaRED substrate was used for antibody detection, and tissue sections were counterstained with Gill’s hematoxylin (all from Vector Laboratories, Burlingame, CA, USA). Positively stained cells from a minimum of four images per tumor were quantified using a Nuance spectral unmixing camera and InForm Tissue Finder software (Perkin Elmer, Waltham, MA, USA).

### 4.12. Statistical Analyses

Band intensities on immunoblots were quantified using Image J [78] and treatments compared to DMSO-treated samples using a Kruskal–Wallis test with Dunn’s multiple comparisons test. Flow cytometry and immunohistochemistry results were compared using a one-way ANOVA with Bonferroni’s correction for multiple comparisons. For in vivo experiments, sample size calculations were performed based on the known mean and standard deviation of survival for the orthotopic implant models. These indicated that with four mice per group, we should be able to detect a true difference in the mean response of treated and control mice of −9.76 or 9.76 days with probability (power) 0.80. The Type I error probability associated with this test of the null hypothesis that the population means of the treated and control groups are equal was 0.05. Kaplan–Meier survival curves were compared using the log-rank (Mantel-Cox) test. THC, CBD, or THC:CBD treated groups were compared to vehicle controls, while THC:CPA and CBD:CPA combination-treated groups were compared to mice treated with CPA alone. Values of significance are indicated by asterisks and described in each figure legend where appropriate.

## 5. Conclusions

Current pre-clinical evidence demonstrates that THC and CBD are effective drugs for adult glioblastoma, both when used in isolation, in combination with each other, or in combination with the conventional treatments temozolomide and radiation [24,26]. Such studies have now led to new clinical trials for adults with brain cancer to determine their clinical benefit. These compounds have also been investigated in a range of different pediatric solid tumors and leukemias [79,80,81]. We aimed to determine if cannabinoids might be effective treatments against medulloblastoma and ependymoma and report here the first study that investigates THC and CBD for the treatment of pediatric brain cancers. Despite promising in vitro data demonstrating that THC and CBD inhibited medulloblastoma and ependymoma cell proliferation, induced cell death in part mediated by the induction of autophagy, and demonstrated synergism when combined with each other, or with conventional chemotherapy to further increase cancer cell death, these results did not translate effectively in vivo and failed to improve animal survival. Pediatric brain cancers are difficult diseases to treat, and it is known that the outcomes for patients with relapsed or refractory disease are dismal. While there is an urgent need for new therapeutics to evaluate for the treatment of these cancers, it is essential that patients enrolling in clinical trials are offered the most promising agents where there is a real chance of clinical benefit, and our study highlights the valuable role that animal models have in the evaluation of potential anti-cancer therapeutics. Overall, while cannabinoids have some cytotoxic activity against medulloblastoma and ependymoma cells in vitro, our in vivo data suggests that the likelihood of children with high-risk brain cancers, such as Group 3 medulloblastoma or *C11orf95* fusion-positive ependymoma, experiencing any clinical benefit from the use of THC or CBD is minimal.

## Data Availability

The data presented in this study are available are available from the European Genome-Phenome Archive (Accession number: EGAS00001004963). Other sequencing data are not publicly available at the time of publication due to pending publication (Ritzmann et al. in preparation) but will be provided upon reasonable request.

## Supplementary Materials

The following are available online at https://www.mdpi.com/2072-6694/13/2/330/s1: Figure S1: Expression of canonical and non-canonical cannabinoid receptors in pediatric brain cancer cell lines, Figure S2: Quantification of immunoblots from Figure 4, Figure S3: Cannabinoids do not improve survival of mice with D425 medulloblastoma, Figure S4: Gating strategy to analyze cell cycle in D283 medulloblastoma cells, Figure S5: Complete cell cycle analyses from Figure 7.
