# Supplementary material for: Assessment of Cannabidiol and Δ9-Tetrahydrocannabiol in Mouse Models of Medulloblastoma and Ependymoma
Source: Cancers (Basel). 2021 Jan 18;13(2):330. doi: 10.3390/cancers13020330 (PMC7829707; doi:10.3390/cancers13020330)
Supplement: Supplementary file 1 [file cancers-13-00330-s001.zip › cancers-1070441-supplementary/Cancers-1070441-research paper WESTERN BLOTS.pdf]

cancers

MDPI

Supplementary information – IMMUNOBLOTS USED IN THE GENERATION OF FIGURES

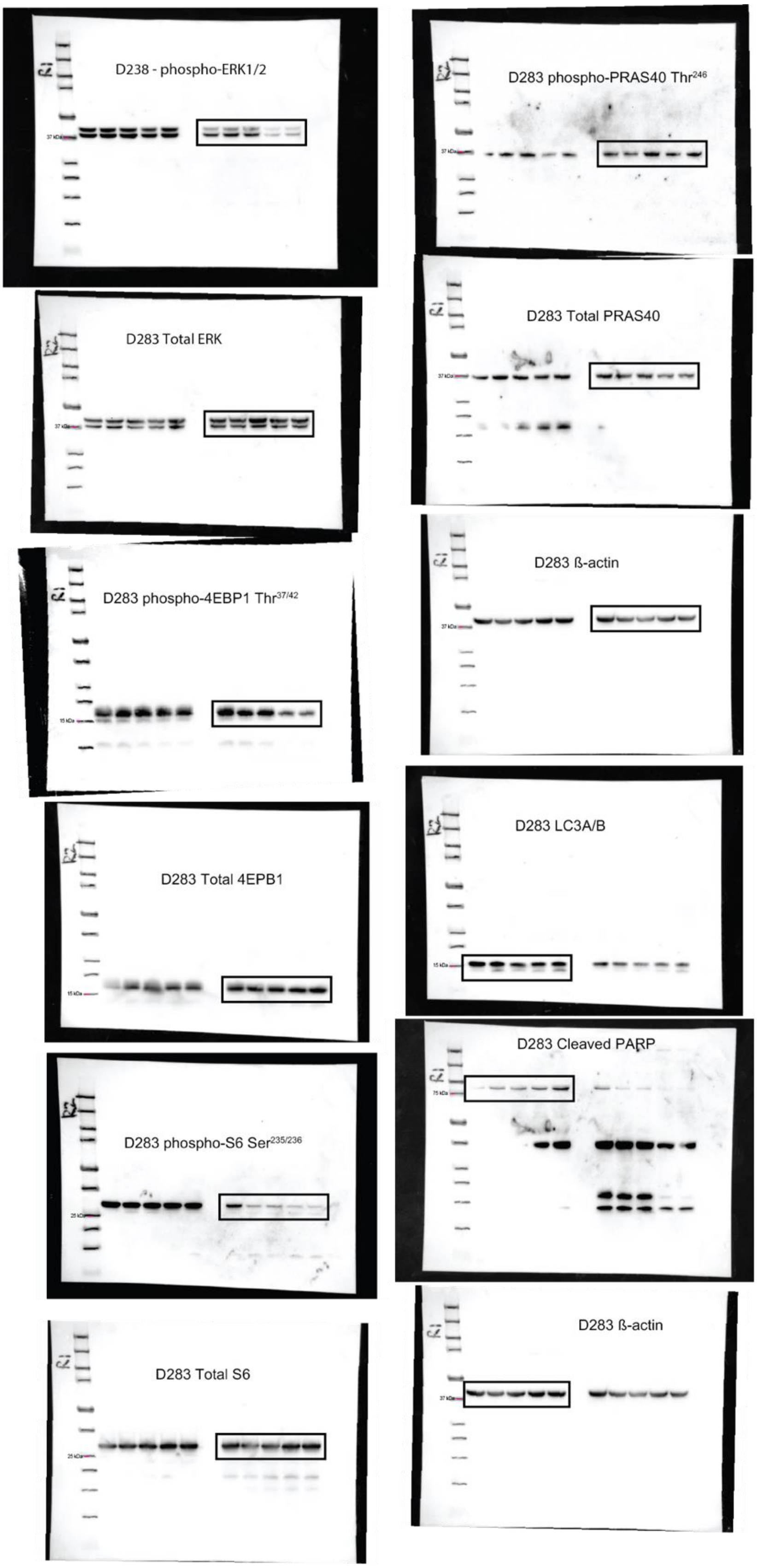

Supplementary figure W1. Immunoblots from D283 medulloblastoma cells treated with THC and CBD. Each membrane is labelled with the antibody target used and the sections of the immunoblot used to generate Figure 4 are indicated by the black box.

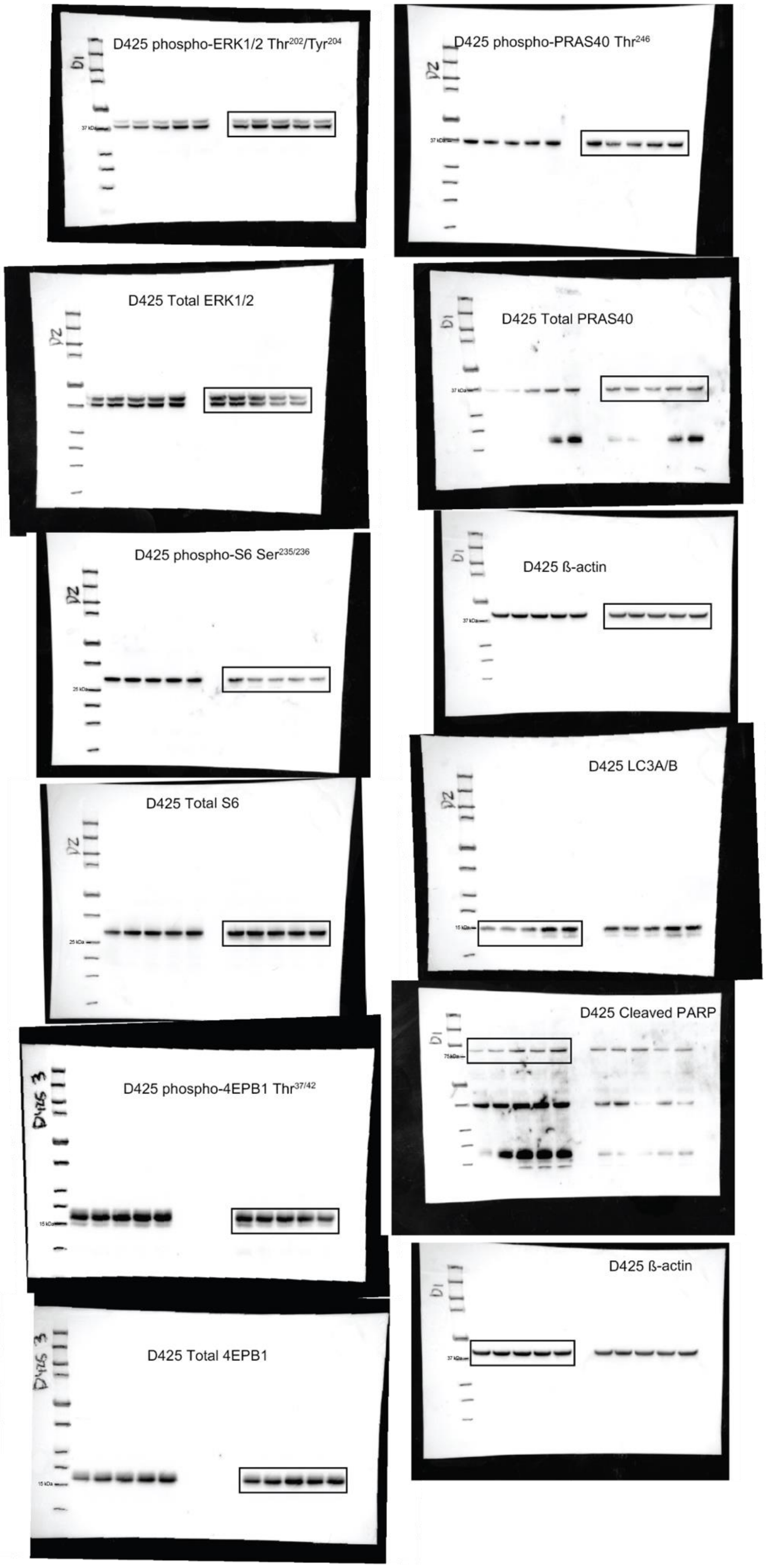

Supplementary figure W2. Immunoblots from D425 medulloblastoma cells treated with THC and CBD. Each membrane is labelled with the antibody target used and the sections of the immunoblot used to generate Figure 4 are indicated by the black box.

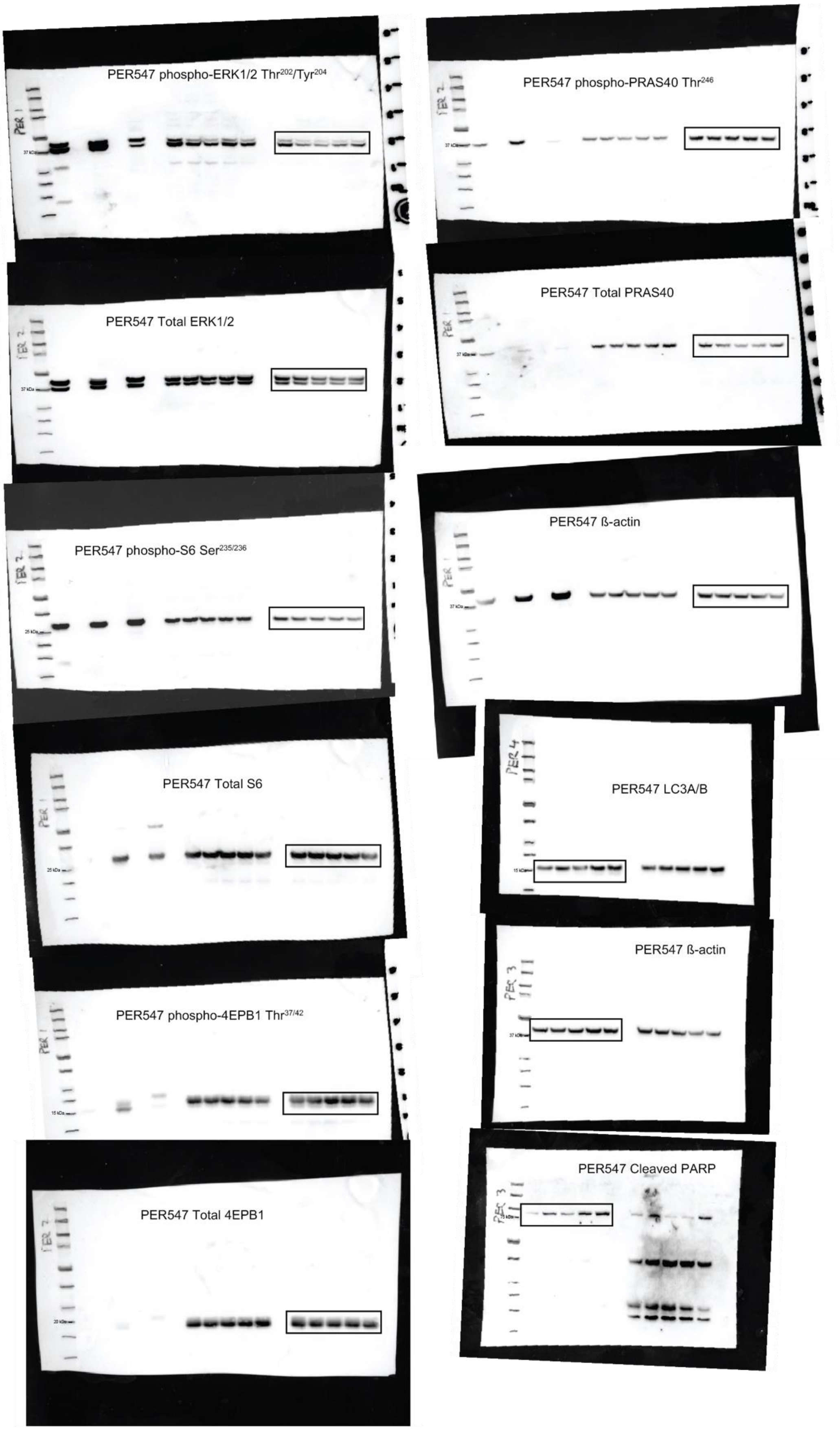

Supplementary figure W3. Immunoblots from PER547 medulloblastoma cells treated with THC and CBD. Each membrane is labelled with the antibody target used and the sections of the immunoblot used to generate Figure 4 are indicated by the black box.
