# Supplementary material for: Assessment of Cannabidiol and Δ9-Tetrahydrocannabiol in Mouse Models of Medulloblastoma and Ependymoma
Source: Cancers (Basel). 2021 Jan 18;13(2):330. doi: 10.3390/cancers13020330 (PMC7829707; doi:10.3390/cancers13020330)
Supplement: Supplementary file 1 [file cancers-13-00330-s001.zip › cancers-1070441-supplementary/cancers-1070441-supplementary.pdf]

cancers

MDPI

Assessment of Cannabidiol and Δ9-Tetrahydrocannabiol in Mouse Models of Medulloblastoma and Ependymoma

Clara Andradas, Jacob Byrne, Mani Kuchibhotla, Mathew Ancliffe, Anya C. Jones, Brooke Carline, Hilary Hii, Alexandra Truong, Lisa C. D. Storer, Timothy A. Ritzmann, Richard G. Grundy, Nicholas G. Gottardo and Raelene Endersby

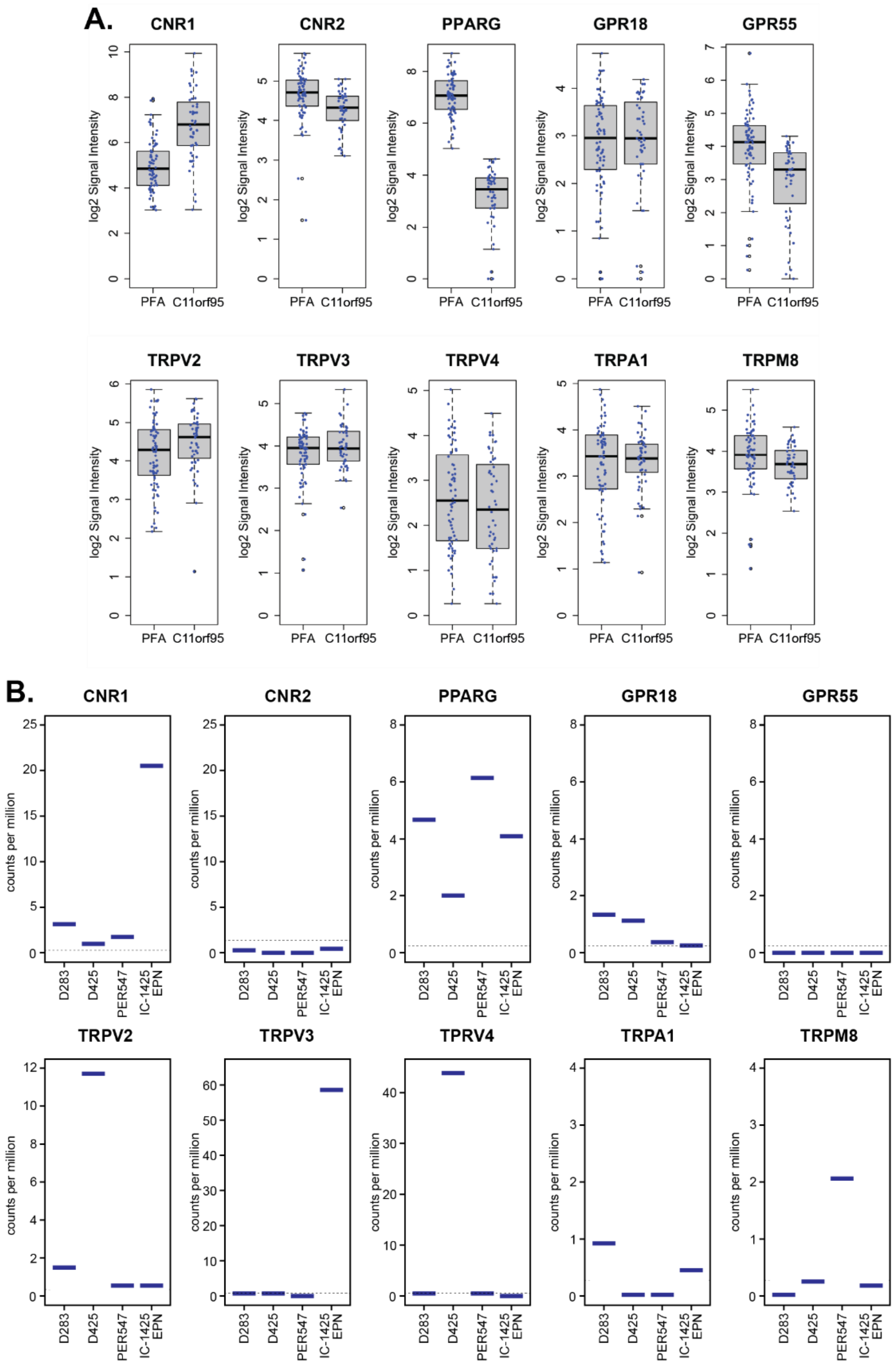

Figure S1. Expression of canonical and non-canonical cannabinoid receptors in pediatric brain cancer cell lines. **(A)** RNAseq data from Figure 1B was validated in an independent microarray dataset of ependymoma samples representing

the molecular subgroups indicated (n=209) [11]. **(B)** Expression of the indicated genes (mean counts per million) was evaluated from four independent RNA-seq datasets from the medulloblastoma cell lines D283, D425 and PER547 and the ependymoma cell line IC-1425EPN.

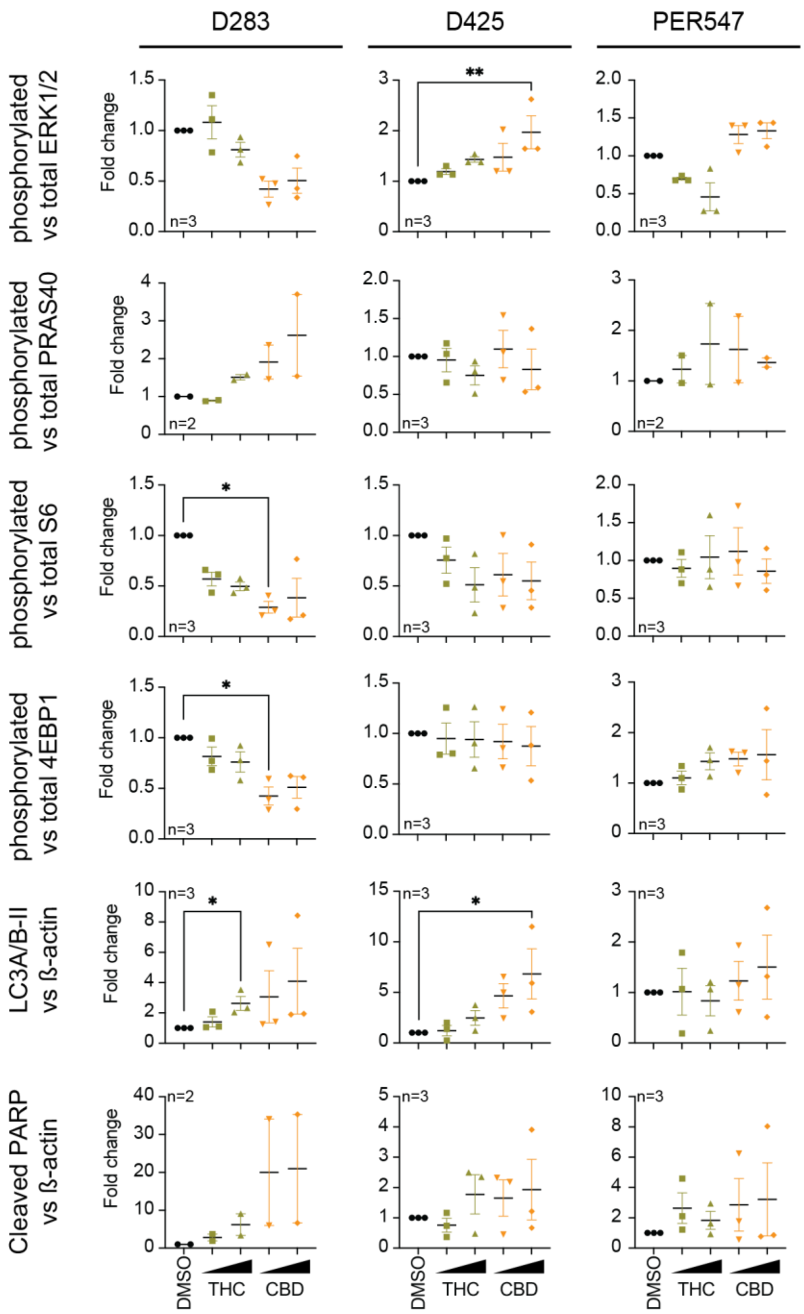

Figure S2. Quantification of immunoblots from Figure 4. Immunoblots presented in Figure 4 from the cell lines (top) and samples (bottom) indicated were quantified using ImageJ software [78]. The triangles indicate the low and high concentrations of THC and CBD as shown in Figure 4. Relative density was calculated by dividing the densitometry of the phosphorylated protein with its respective total protein, or for LC3-II and cleaved PARP by comparing the protein with β-actin. Data are presented as fold change of the respective DMSO-treated sample. The number of independent experiments performed are indicated (n). Drug-treated samples were compared to the DMSO-treated controls using a Kruskal-Wallis test with Dunn's multiple comparisons test (*, p<0.0332; **, p<0.0021).

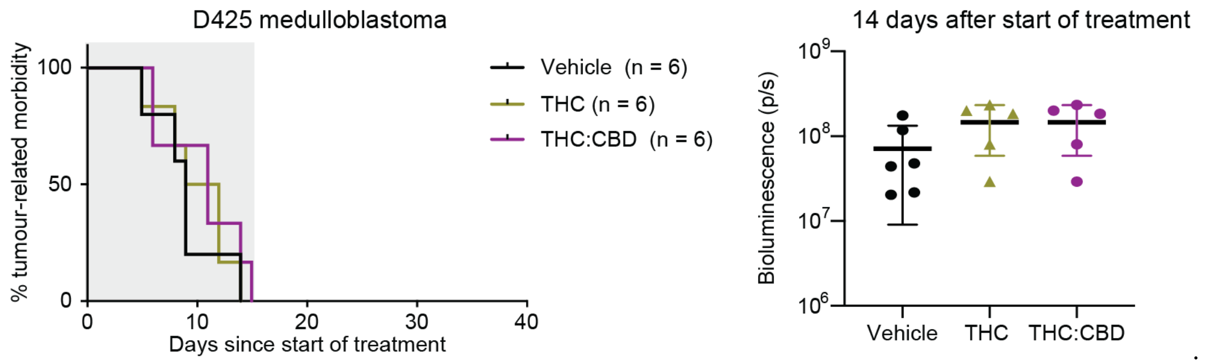

Figure S3. Cannabinoids do not improve survival of mice with D425 medulloblastoma. Mice with orthotopic D425 medulloblastoma xenografts were established and treated with vehicle, 45 mg/kg THC or a 1:1 combination of 22.5 mg/kg THC with 22.5 mg/kg CBD. Kaplan-Meier survival analyses show that no significant difference in survival were observed following cannabinoid treatment. Comparisons of the bioluminescence detected in mice from each treatment groups confirmed that no difference in tumor burden was observed.

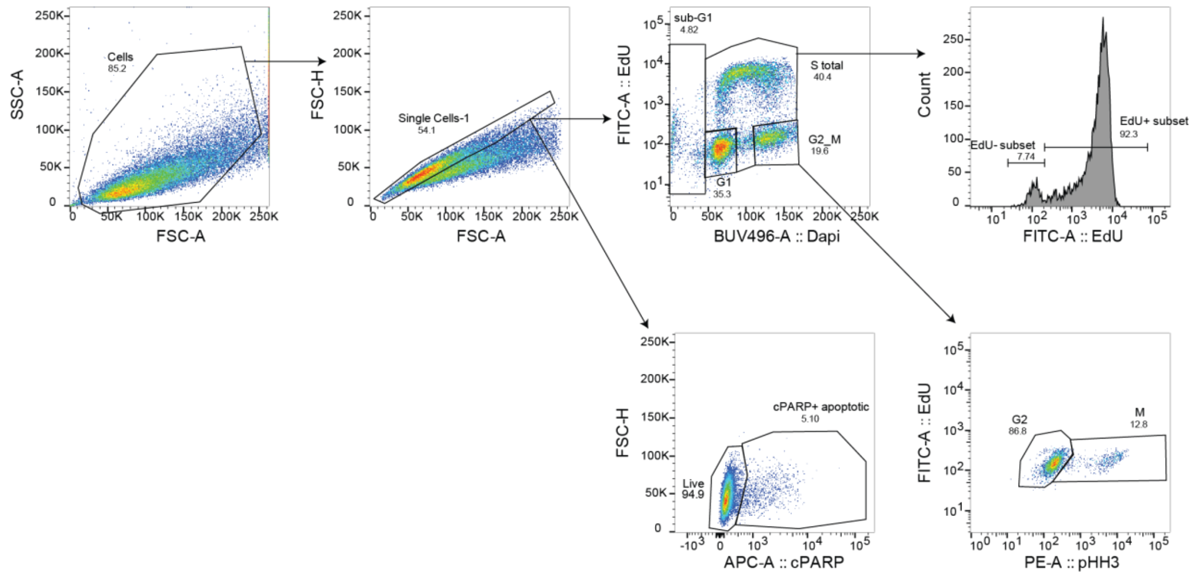

Figure S4. Gating strategy to analyse cell cycle in D283 medulloblastoma cells. Single cells were isolated using forward scatter (FCS) and side scatter (SSC) parameters, followed by FSC area and height parameters. Single cells were separated into sub-G1, G1, S and G2/M populations using DAPI and EdU. Cells in S phases were further sub divided into those in S phase arrest (EdU negative) or those actively synthesizing DNA (EdU positive). Cells in mitosis were identified as positive for phosphorylated histone H3 (pHH3) from the G2/M gate. The proportion of cells undergoing apoptosis (positive for cleaved (c) PARP) was determined from the single cell gate.

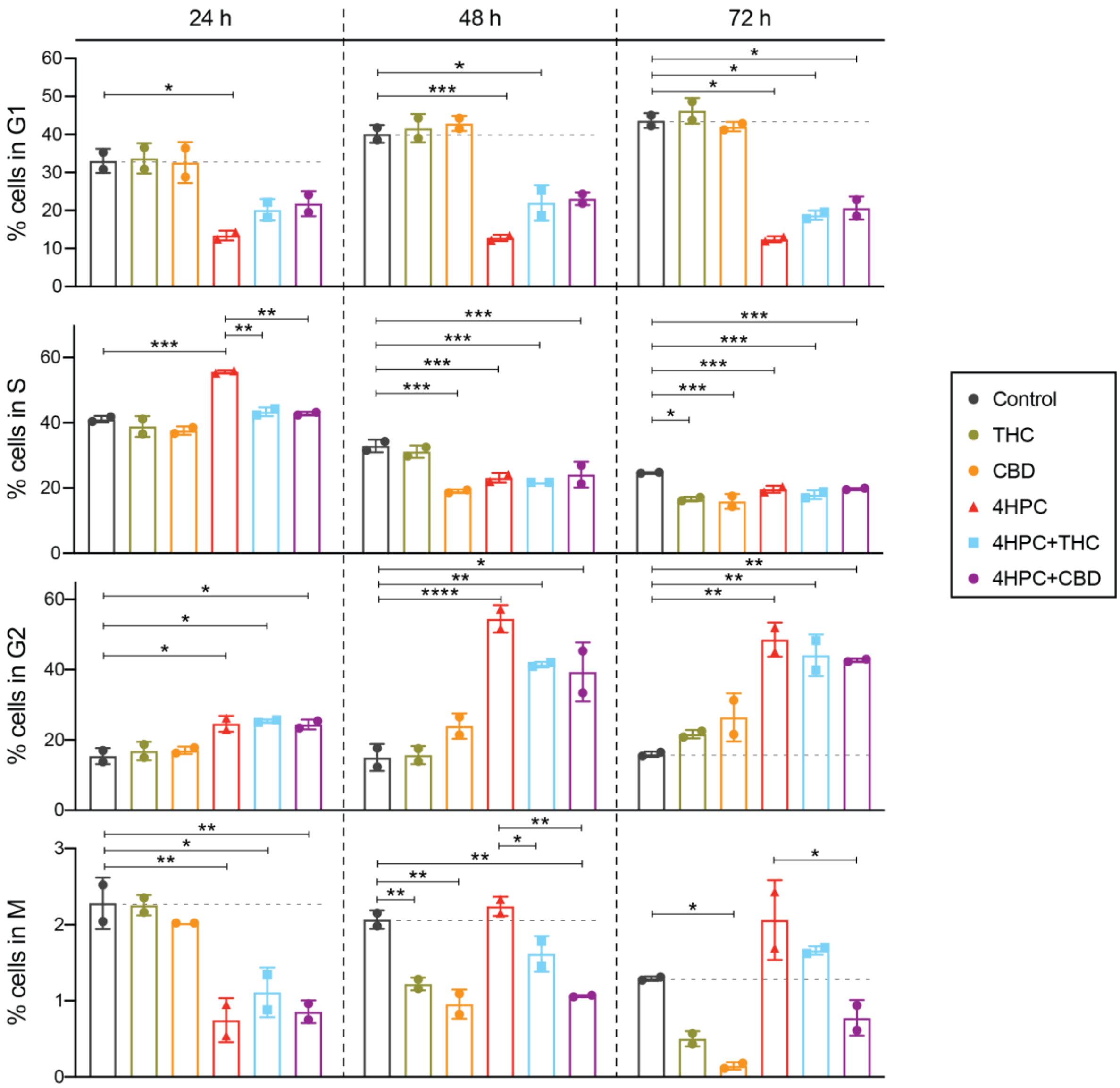

Figure S5. Complete cell cycle analyses from Figure 7. D283 medulloblastoma cells were treated with DMSO, THC, or CBD in the presence of DMSO or activated CPA (4HPC) and cell cycle distribution was assessed using flow cytometry. Different cell cycle phases were determined as described in Supplementary Figure 4 and data from two independent experiments were quantified. Shown are mean ± SD for cells in G1, S phase, G2 or M. The effects of THC and CBD were determined by comparing drug treated samples with DMSO-treated controls (black bars), or combination treated samples with cells treated with 4HPC alone (red bars). Statistical significance was established using one-way ANOVA with Bonferroni's correction for multiple comparisons (*, $p<0.033$; **, $p<0.0021$; ***, $p<0.0002$; ****, $p<0.0001$).
